# Time Patterns in Internal Human Exposure Data to Bisphenols, Phthalates, DINCH, Organophosphate Flame Retardants, Cadmium and Polyaromatic Hydrocarbons in Europe

**DOI:** 10.3390/toxics11100819

**Published:** 2023-09-28

**Authors:** Laura Rodriguez Martin, Liese Gilles, Emilie Helte, Agneta Åkesson, Jonas Tägt, Adrian Covaci, Amrit K. Sakhi, An Van Nieuwenhuyse, Andromachi Katsonouri, Anna-Maria Andersson, Arno C. Gutleb, Beata Janasik, Brice Appenzeller, Catherine Gabriel, Cathrine Thomsen, Darja Mazej, Denis Sarigiannis, Elena Anastasi, Fabio Barbone, Hanna Tolonen, Hanne Frederiksen, Jana Klanova, Jani Koponen, Janja Snoj Tratnik, Kim Pack, Koppen Gudrun, Kristin Ólafsdóttir, Lisbeth E. Knudsen, Loïc Rambaud, Loreta Strumylaite, Lubica Palkovicova Murinova, Lucia Fabelova, Margaux Riou, Marika Berglund, Maté Szabados, Medea Imboden, Michelle Laeremans, Milada Eštóková, Natasa Janev Holcer, Nicole Probst-Hensch, Nicole Vodrazkova, Nina Vogel, Pavel Piler, Phillipp Schmidt, Rosa Lange, Sónia Namorado, Szilvia Kozepesy, Tamás Szigeti, Thorhallur I. Halldorsson, Till Weber, Tina Kold Jensen, Valentina Rosolen, Vladimira Puklova, Wojciech Wasowicz, Ovnair Sepai, Lorraine Stewart, Marike Kolossa-Gehring, Marta Esteban-López, Argelia Castaño, Jos Bessems, Greet Schoeters, Eva Govarts

**Affiliations:** 1 VITO Health, Flemish Institute for Technological Research (VITO), 2400 Mol, Belgium; liese.gilles@vito.be (L.G.); gudrun.koppen@vito.be (K.G.); michelle.laeremans@vito.be (M.L.); jos.bessems@vito.be (J.B.); greet.schoeters@uantwerpen.be (G.S.); eva.govarts@vito.be (E.G.); 2 Institute of Environmental Medicine, Karolinska Institutet, 17177 Stockholm, Sweden; emilie.helte@ki.se (E.H.); agneta.akesson@ki.se (A.Å.); jonas.tagt@ki.se (J.T.); marika.berglund@ki.se (M.B.); 3 Toxicological Centre, University of Antwerp, Universiteitsplein 1, 2610 Antwerp, Belgium; adrian.covaci@uantwerpen.be; 4 Norwegian Institute of Public Health, 0456 Oslo, Norway; amritkaur.sakhi@fhi.no (A.K.S.); cathrine.thomsen@fhi.no (C.T.); 5 Laboratoire National de Santé (LNS), Rue Louis Rech 1, 3555 Dudelange, Luxembourg; an.vannieuwenhuyse@lns.etat.lu; 6 State General Laboratory, Ministry of Health, 2081 Nicosia, Cyprus; akatsonouri@sgl.moh.gov.cy (A.K.); eanastasi@sgl.moh.gov.cy (E.A.); 7 Department of Growth and Reproduction, Copenhagen University Hospital, Rigshospitalet, 2100 Copenhagen, Denmark; anna-maria.andersson@regionh.dk (A.-M.A.); hanne.frederiksen@regionh.dk (H.F.); 8 International Center for Research and Research Training in Endocrine Disruption of Male Reproduction and Child Health (EDMaRC), University of Copenhagen, Rigshospitalet, 2100 Copenhagen, Denmark; 9 Luxembourg Institute of Science and Technology (LIST), 4362 Esch-sur-Alzette, Luxembourg; arno.gutleb@list.lu; 10 Nofer Institute of Occupational Medicine, 91-348 Lodz, Poland; beata.janasik@imp.lodz.pl (B.J.); wojciech-wasowicz@wp.pl (W.W.); 11 Luxembourg Institute of Health (LIH), 1445 Strassen, Luxembourg; brice.appenzeller@lih.lu; 12 Environmental Engineering Laboratory, Department of Chemical Engineering, Aristotle University of Thessaloniki, 54124 Thessaloniki, Greece; katerinagabriel79@gmail.com (C.G.); sarigiannis@auth.gr (D.S.); 13 HERACLES Research Center on the Exposome and Health, Center for Interdisciplinary Research and Innovation, Balkan Center, Bldg. B, 10th km Thessaloniki-Thermi Road, 57001 Thessaloniki, Greece; 14 Jožef Stefan Institute, 1000 Ljubljana, Slovenia; darja.mazej@ijs.si (D.M.); janja.tratnik@ijs.si (J.S.T.); 15 Environmental Health Engineering, Institute of Advanced Study, Palazzo del Broletto–Piazza Della Vittoria 15, 27100 Pavia, Italy; 16 Department of Medicine, Surgery and Health Sciences, University of Trieste, Strada di Fiume, 447, 34149 Trieste, Italy; fabio.barbone@uniud.it; 17 Finnish Institute for Health and Welfare (THL), 00271 Helsinki, Finland; hanna.tolonen@thl.fi (H.T.); jani.koponen@thl.fi (J.K.); 18 RECETOX, Faculty of Science, Masaryk University, Kotlarska 2, 625 00 Brno, Czech Republic; jana.klanova@recetox.muni.cz (J.K.); pavel.piler@recetox.muni.cz (P.P.); 19 Department of Toxicology, Health-Related Environmental Monitoring, German Environment Agency (UBA), 14195 Berlin, Germany; kim.pack@uba.de (K.P.); nina.vogel@uba.de (N.V.); phillipp.schmidt@uba.de (P.S.); rosa.lange@uba.de (R.L.); till.weber@uba.de (T.W.);; 20 Faculty of Food Science and Nutrition, University of Iceland, Hofsvallagata 53, 107 Reykjavik, Iceland; stinaola@hi.is (K.Ó.); tih@hi.is (T.I.H.); 21 Section of Environmental Health, University of Copenhagen, 1165 Copenhagen, Denmark; liek@sund.ku.dk; 22 Department of Environmental and Occupational Health, Santé Publique France, 94410 Saint Maurice, Francemargaux.riou@santepubliquefrance.fr (M.R.); 23 Neuroscience Institute, Medical Academy, Lithuanian University of Health Sciences, LT-50161 Kaunas, Lithuania; loreta.strumylaite@lsmuni.lt; 24 Department of Environmental Medicine, Faculty of Public Health, Slovak Medical University, 833 03 Bratislava, Slovakia; lubica.murinova@szu.sk (L.P.M.);; 25 National Public Health Center, Albert Florian 2-6, 1097 Budapest, Hungary; szabados.mate@nnk.gov.hu (M.S.); kozepesy.szilvia@nnk.gov.hu (S.K.); szigeti.tamas@nnk.gov.hu (T.S.); 26 Swiss Tropical and Public Health Institute, Kreuzstrasse 2, 4123 Allschwil, Switzerland; medea.imboden@swisstph.ch (M.I.); nicole.probst@swisstph.ch (N.P.-H.); 27 Department of Environment and Health, Public Health Authority, 83105 Bratislava, Slovakia; milada.estokova@uvzsr.sk; 28 Division for Environmental Health, Croatian Institute of Public Health, Rockefellerova 7, 10000 Zagreb, Croatia; natasa.janev@hzjz.hr; 29 Department of Social Medicine and Epidemiology, Faculty of Medicine, University of Rijeka, Bráce Branchetta 20/1, 51000 Rijeka, Croatia; 30 Centre for Health and Environment, National Institute of Public Health, 100 00 Prague, Czech Republic; nicole.vodrazkova@szu.cz (N.V.); vladimira.puklova@szu.cz (V.P.); 31 Department of Epidemiology, National Institute of Health Doctor Ricardo Jorge, Avenida Padre Cruz, 1649-016 Lisbon, Portugal; sonia.namorado@insa.min-saude.pt; 32 Department of Clinical Pharmacology, Pharmacy and Environmental Medicine, University of Southern Denmark, 5000 Odense, Denmark; tkjensen@health.sdu.dk; 33 Central Directorate for Health, Social Policies and Disability, Friuli Venezia Giulia Region, Via Cassa di Risparmio 10, 34121 Trieste, Italy; valentina.rosolen@regione.fvg.it; 34 UKHSA UK Health Security Agency, Harwell Science Park, Chilton OX11 0RQ, UK; ovnair.sepai@ukhsa.gov.uk (O.S.); lorraine.stewart@ukhsa.gov.uk (L.S.); 35 National Centre for Environmental Health, Instituto de Salud Carlos III, 28220 Majadahonda, Spain; m.esteban@isciii.es (M.E.-L.); castano@isciii.es (A.C.)

**Keywords:** human biomonitoring, hazardous chemical, phthalates, DINCH, OPFRs, cadmium, PAHs, bisphenols

## Abstract

Human biomonitoring (HBM) data in Europe are often fragmented and collected in different EU countries and sampling periods. Exposure levels for children and adult women in Europe were evaluated over time. For the period 2000–2010, literature and aggregated data were collected in a harmonized way across studies. Between 2011–2012, biobanked samples from the DEMOCOPHES project were used. For 2014–2021, HBM data were generated within the HBM4EU Aligned Studies. Time patterns on internal exposure were evaluated visually and statistically using the 50th and 90th percentiles (P50/P90) for phthalates/DINCH and organophosphorus flame retardants (OPFRs) in children (5–12 years), and cadmium, bisphenols and polycyclic aromatic hydrocarbons (PAHs) in women (24–52 years). Restricted phthalate metabolites show decreasing patterns for children. Phthalate substitute, DINCH, shows a non-significant increasing pattern. For OPFRs, no trends were statistically significant. For women, BPA shows a clear decreasing pattern, while substitutes BPF and BPS show an increasing pattern coinciding with the BPA restrictions introduced. No clear patterns are observed for PAHs or cadmium. Although the causal relations were not studied as such, exposure levels to chemicals restricted at EU level visually decreased, while the levels for some of their substitutes increased. The results support policy efficacy monitoring and the policy-supportive role played by HBM.

## 1. Introduction

The European Human Biomonitoring Initiative [1] (HBM4EU) was a joint effort by 30 countries and the European Environmental Agency operating at the science–policy interface, cofounded under the European Commission’s Horizon 2020 program (2017–2021). The goal of HBM4EU was to monitor the internal exposure of the general population in Europe to a variety of prioritized chemical pollutants, to evaluate their possible health impacts and to generate relevant science-based input to support policy making by combining, harmonizing and analyzing data from EU national and regional studies.

After several rounds of prioritization, according to a structured protocol, chemical substance groups were selected for which new knowledge was generated to answer specific policy questions. One of the questions was the identification of time patterns as a tool to evaluate the efficacy of established policies to reduce the exposure of European citizens to chemicals and as an early warning for rising concentrations of hazardous chemicals in the European population. Chemicals were prioritized with input from the EU and national policy makers, risk assessors and a stakeholder forum. They considered both national and EU level policy needs to better understand chemical exposure and health outcomes [1]. This first prioritization round resulted in nine chemical groups, i.e., bisphenols, cadmium (Cd), polycyclic aromatic hydrocarbons (PAHs), phthalates and DINCH, per- and polyfluoroalkyl substances (PFAS), flame retardants (FR), chemical mixtures, anilines and emerging chemicals.

Within the HBM4EU, a HBM survey was conducted in 23 countries to generate EU-wide comparable HBM data, namely the HBM4EU Aligned Studies [2,3] in children, adults and teenagers between 2014 and 2021. The HBM4EU aligned studies followed the DEMOCOPHES feasibility project, which was the first project to perform harmonized HBM at the European scale with samples from children and their mothers between 2011 and 2012 [4]. To investigate the time patterns, substance groups and target populations that were overlapping between the HBM4EU Aligned Studies and DEMOCOPHES were considered. The chemical groups are phthalates, DINCH and organophosphate flame retardants (OPFRs) in children and Cd, bisphenols and PAHs in adult women.

Phthalates are synthetic organic chemicals mostly used as plasticizers in plastics or in personal care products [5], but as they are not chemically bound to the materials they are added to, they can easily leach out or evaporate over time. Routes of exposure include ingestion, inhalation and skin absorption [6]. Several phthalates are endocrine-disrupting chemicals, classified by the European Chemicals Agency (ECHA) as substances of very high concern (SVHCs) [7]. Exposure to phthalates has an impact on the respiratory, reproductive, cardiovascular and immune systems [6], and exposure during childhood might affect normal development and increase the risk of reproductive and allergic diseases [8]. DINCH was introduced to the EU market in 2002 as a substitute for phthalates and has been increasingly used in certain manufacturing products [9] as a replacement alternative compound. DINCH nephrotoxicity has been studied in rat models, where it has been shown to cause impairments in metabolic pathways and hormone production, which might affect the reproductive system [10].

Flame retardants are substances added to a variety of consumer products to reduce flammability and ensure compliance with government regulations on fire safety. Polybrominated diphenyl ethers (PBDEs) were the most used flame retardants until they had to be substituted by other chemicals due to their persistent bioaccumulation and adverse health effects [11], which is why they were banned under the Stockholm Convention on Persistent Organic Pollutants [12]. Alternative replacements for PBDEs include organophosphate flame retardants (OPFRs), and halogenated and non-halogenated additive substances, which have been detected in indoor dust, baby products, hand wipes and other household products [13]. The main route of exposure for toddlers is dust ingestion and air inhalation for adults [14]. Children present higher levels of exposure to OPFRs, probably due to being closer to accumulated dust on the floor, hand-to-mouth activity and their higher body surface area to internal mass ratio. Recent studies in humans have also shown associations between OPFRs exposure and behavioral problems and impaired cognitive performance in children [15].

Cadmium (Cd) is a ubiquitous toxic metal. Its main exposure sources for humans include polluted soil and air near industry, contamination through the use of phosphate fertilizers and/or food grown in contaminated soil, and smoking. The main intake route for the non-smoker population is diet. Its adverse health effects are multiple, including renal and bone damage and cancer risk, even at a low level of exposure [16]. Cd has been classified by the International Agency for Research on Cancer (IARC) as a Group I human carcinogen.

Bisphenol A (BPA) is a phenolic organic chemical compound whose exposure can originate from different sources, the most important being consumption of contaminated food and beverages [17]. BPA is an endocrine disruptor associated with several adverse effects. In women of reproductive age, effects on female reproduction [18] and on the immune system at very low levels of exposure have been reported [19]. In pregnant women, BPA can cause miscarriages and premature deliveries [17]. BPA was added by the ECHA to the candidate list of substances of very high concern in 2017 [20], causing its replacement in recent years by other bisphenols with a similar structure and properties, such as bisphenol F (BPF) and bisphenol S (BPS) in common products, such as thermal paper [21]. These substitutes are less studied but might pose similar health risks [22].

Polycyclic aromatic hydrocarbons (PAHs) are ubiquitous in the environment due to the combustion of fossil fuels and organic waste. Additionally, a typical source of PAHs for smokers and people in their environment is tobacco smoke. Several PAHs are considered by the IARC as known or possible carcinogens, and to have other non-carcinogenic effects in the pulmonary, gastrointestinal, renal and dermatologic systems [23].

Time patterns for the selected priority substances have, to our knowledge, been partially explored at country level and only to a very limited extent at the European scale. Chemicals are regulated under REACH at the EU level, but enforcement remains at the national level. Furthermore, the environment, habits and lifestyle within Europe varies between countries and may influence exposure levels. Therefore, the aim of this study is to provide insight into the changes in the internal exposure of the general population in Europe, specifically adult women (24 to 52 years old) and children (5 to 12 years old), to chemicals belonging to the selected priority chemical groups, and to explore whether their biomarker levels decrease, increase or remain stable over time. Furthermore, we would like to evaluate whether introducing regulatory measures in the legislation has influenced the patterns in the exposure levels observed over time.

## 2. Materials and Methods

### 2.1. Study Design and Study Population

Three sampling time periods were selected to explore the time patterns in the internal exposure to the European population, making use of the European HBM4EU Aligned Studies within HBM4EU (2014–2021) [2,24,25], the DEMOCOPHES project (2011–2012) [4] and other available HBM studies within the time period 2000–2010. Two age groups were considered: (1) adult women between 24 and 52 years old and (2) children between 5 and 12 years old. The age groups were based on the available ages/sexes within the DEMOCOPHES project and the HBM4EU Aligned Studies. The ages for the rest of the studies are indicated in Supplementary Tables S1–S6. The following chemical groups were selected: phthalates, DINCH and OPFRs for children; Cd, bisphenols and PAHs for adult women. The chemical groups were chosen based on being the 1st set of prioritized substance groups measured for the respective age groups in the overlapping DEMOCOPHES and HBM4EU Aligned Studies population. As the DEMOCOPHES project measured biomarkers belonging to the first set of prioritized substances in urine, this matrix was the only one selected. Furthermore, biomarkers were selected based on the overlap of the available biomarkers from the DEMOCOPHES and Aligned Studies projects. Data for each time period were collected using different strategies.

For the first time period (2000–2010), studies that fulfilled the following inclusion criteria were selected: (i) the sampling years were between 2000 and 2010, (ii) the population group (general population) and age ranges were the same as in the DEMOCOPHES and the HBM4EU Aligned Studies, that is, 5–12 years old for children and 24 to 52 years old for adult women, (iii) the availability of urinary biomarkers belonging to the first set of priority chemical groups measured in the HBM4EU Aligned Studies for those age groups, and (iv) the availability of P50 or P90 statistical values. First, harmonized aggregated HBM data made accessible by the HBM4EU partners [26] through the European HBM dashboard (https://hbm.vito.be/eu-hbm-dashboard, accessed on 1 August 2023) were included if the inclusion criteria were fulfilled. Data harmonization included transforming the study’s specific variables into a common set of variables defined by a basic codebook and converting all toxicological samples into the same units. Additionally, extensive literature research was performed in PubMed to identify other published HBM data for the time period, with the language and date restriction set to the English or Swedish language, published in 2000 or later. The following search terms were used: (1) “DINCH” in combination with “urine” or “Human Biomonitoring” for “DINCH”, (2) “1-hydroxynaphthalene”, “2-hydroxynaphthalene”, “1-hydroxypyrene” or “PAH” in combination with “urine” or “Human Biomonitoring” for PAHs, (3) “bisphenol” in combination with “urine” or “Human Biomonitoring” for bisphenols, (4) “organophosphate flame retardants” or “diphenyl phosphate” in combination with “urine” or “Human Biomonitoring” for OPFRs, (5) “phthalate” or “phthalates” in combination with “urine” or “Human Biomonitoring” for phthalates, and (6) “cadmium” in combination with “urine” or “Human Biomonitoring” for cadmium.

The inclusion criteria were fitted as much as possible to points (i), (ii), (iii) and (iv), considering that the data were collected from publications and, thus, the selection process needed to be less restrictive to include as much data as possible. This implied that, for some studies, the age ranges might be slightly outside 24–52 years old for women and 5–12 years old for children, or that no stratification was performed for sex and, therefore, male participants were included in the calculation for the P50 and P90 values. No minimum sample size was imposed to include as many studies for the time point as possible.

For the second time period (2011–2012), data from the DEMOCOPHES project was considered. This was the first European scale HBM survey [4], which generated comparable HBM data from 17 EU countries. For 12 DEMOCOPHES countries, the data were harmonized using the same harmonization tools and aggregation scripts as the harmonized aggregated data for the first time period. Additionally, for the 5 remaining DEMOCOPHES countries, the data were extracted from publications: phthalate and cadmium data for Switzerland, Ireland, Portugal, Romania and the United Kingdom [4].

The third and most recent time period (2014–2021) was based on the HBM4EU Aligned Studies [2,24,25], in which 23 countries generated EU-wide comparable HBM data. A total number of 10,705 participants were recruited between 2014 to 2021, separated into three age groups: (i) 3576 children aged 6–12 years, (ii) 3117 teenagers aged 12–18 years and (iii) 4102 young adults aged 20–39 years. Moreover, 11 to 12 countries were included per age group and the population was geographically distributed over Europe.

Supplementary Tables S1–S6 show the study characteristics and the available stratified data for each of the chemical groups for each of the three time points, as they were used in the analysis.

### 2.2. Exposure Analysis

The following chemical groups were analyzed in urine: phthalates [27] and the substitute DINCH, halogenated and organophosphorus flame retardants [28] for children, and cadmium, bisphenols [29] and polycyclic aromatic hydrocarbons (PAHs) for adult women.

For some DEMOCOPHES countries, the biobanked samples were further analyzed for the first set of chemical groups prioritized within HBM4EU using stringent quality control programs for chemical analysis. The original DEMOCOPHES data were accessed (i.e., phthalates, Cd and bisphenols for some countries [30]) and, for other countries, additional analysis was performed on the biobanked samples to generate new exposure data, specifically DINCH for Denmark; BPA, DINCH, phthalates and PAHs for Poland; DINCH for Spain; BFP, BPS, OPFRs, DINCH and PAHs for Sweden; bisphenols, PAHs, phthalates and DINCH for Luxembourg; DINCH, PAHs and OPFRs for Germany; OPFRs, phthalates, DINCH, bisphenols and PAHs for the Czech Republic; OPFRs, phthalates, DINCH and bisphenols for Belgium; and OPFRs, phthalates, DINCH, bisphenols and PAHs for Cyprus. If the biomarkers were remeasured, the oldest measurement was used for comparability reasons with those countries without remeasured samples, except for Cyprus where the remeasured biomarkers were used, as the study leader had indications that the remeasured 2020 results were more robust from an analytical point of view. To ensure that the sample integrity had not being compromised, the creatinine values were also remeasured and excellent correspondence with the values from 2012 vs. 2020 was found.

In Supplementary Table S7, the analytical methods used to measure all the substance groups for all the studies are listed. For the first time period, no information regarding the quality or comparability of the analytical results for the different identified studies was found, as the data were obtained directly from publications that did not specify this information.

Within the DEMOCOPHES project, a quality assurance program was established to guarantee the quality and comparability of the analytical results among laboratories, as described by Schindler et al. [31]. Standard operating procedures (SOPs) were developed, and all laboratories received and were compliant with them. Furthermore, interlaboratory comparison investigations (ICIs) and external quality assessment schemes (EQUAS) were organized and evaluated for all participating laboratories and the successful ones were labelled as “qualified laboratories” [32].

The comparability, reliability and quality of the samples from the HBM4EU Aligned Studies and the newly measured biobanked samples from the participating DEMOCOPHES studies was ensured by enforcing the sample analysis in the laboratories participating in the ICI/EQUAS, organized as part of the HBM4EU initiative, which is described by Esteban et al. [32], and, in some specific papers, for each of the substance groups analyzed [26,27,28,29,33,34].

### 2.3. Statistical Methods

Summary statistics for the first time period harmonized data were used; the second and third time periods were calculated in a harmonized way using the R script developed in the HBM4EU project for the harmonized aggregated data between 2000–2010, 12 of the DEMOCOPHES studies and the HBM4EU Aligned Studies, to ensure that the data manipulations were performed following the same strategies, such as creatinine standardization and percentile calculation.

For the first time period, summary statistics, i.e., the 50th and 90th percentile (P50 and P90), were retrieved from the literature for the studies sampled between 2000–2010 and for 5 of the DEMOCOPHES studies that did not participate in sharing harmonized aggregated data during the HBM4EU project.

The time pattern analysis was performed on the biomarker data, standardized per gram creatinine to account for sample type differences (first morning urine, random spot and 24 h urine) between the studies.

The summary statistics retrieved from the publications varied among the studies in regard to the percentiles provided, the measurement units, the age group and the strata for which the percentiles were available. In a number of cases, the data had to be further standardized to be comparable with the harmonized aggregated data obtained through the R script. The literature data provided in μg/L were transformed into μg/g creatinine by dividing by an average urinary creatinine concentration of 120 mg/dL [35].

The calculation on the harmonized aggregated data was performed according to a standardized procedure developed during the HBM4EU project. Instructions were given to the HBM4EU partners to harmonize their data according to a basic codebook, which improved data comparability. An R script was developed to calculate the aggregated data using the harmonized individual data. Summary statistics were provided for the complete study, but also for stratified data, depending on the collected information, such as sex, age, educational level or smoking status stratified summary statistics. Additionally, the summary statistics were calculated for volume-based units (µg/L) and creatinine standardized units (µg/g crt). Percentiles were calculated using the generic function ‘quantile’ in the R ‘stats’ package (version 3.6.2), which produces sample quantiles corresponding to the given probabilities.

The summary statistics from the obtained harmonized HBM data are visualized in the European HBM dashboard [26], developed within the HBM4EU project.

To assess time patterns in exposure, visual representation and statistical tests were performed on those markers, per substance group, available in at least two time periods and with at least three data points for the P50 value or three data points for the P90 value for each of the time periods. Sensitivity analyses were performed including all the data and without outliers.

For the visual representation, the percentiles per study were represented in X-Y plots, the *X*-axis being the year when the study started sampling and the *Y*-axis the observed percentile of exposure, conducted separately for the P50 and P90 values. Geometric smooth lines based on polynomial regression fitting were added for visualization purposes to the overall data. Additionally, vertical lines were added representing those moments in time when regulations were established at European or national level.

To assess whether there were any statistically significant changes/patterns in the internal exposure to different biomarkers over time, the Theil–Sen regression estimator was applied. The analysis was performed in R Studio (R version 4.1.3 (10 March 2022)) and the mblm (median-based linear models) package was used to perform linear models based on the Theil–Sen single median. Theil–Sen regression was selected due to the low number of observations (one 50th and/or 90th percentile value per study), as it is a non-parametric approach to linear models. Furthermore, it provides a robust estimate of the slope and trend, as it is a method unbiased by the presence of outliers. The percentile value (50th or 90th) was used as a dependent variable and the sampling year was used as an independent variable when constructing the models. To account for differences in the sample size in the different studies, relative weights were applied to the data as a number of repetitions of each data point. For the weight calculation, a minimum sample size of 120 was proposed earlier by Poulsen et al. [36] to obtain percentiles with reasonably narrow confidence intervals. Taking this value as a reference, the percentiles derived from a study with N equal or bigger than 120 were considered as fully representative and, therefore, assigned a weight equal to 1. For studies with a smaller sample size, a relative weight was calculated as N/120, rounded up. The number of repetitions for one observation was then calculated as the relative weight times ten. This means, the points with full weight (1 = 100%) were used 10 times, and the points with lower weight 1 (weight < 0.15) to 10 (weight > 0.9) times were within the Theil–Sen estimator.

## 3. Results and Discussion

### 3.1. Time Patterns Observed in Children (5–12 Years) in Europe

#### 3.1.1. Phthalates and DINCH

For phthalates, aggregated data over 11 studies for the first time period, 17 studies for the second time period and 12 studies for the third time period were used in the visualization and statistical analysis. Phthalates is the substance group for which the most biomarkers were measured in this work and for which the most regulations have been established in the last decade.

In 2005, the ban from 1999 to prohibit the use of phthalates in children’s toys was made permanent. Three phthalates were banned for use in soft PVC toys and childcare articles (di(2-ethylhexyl) phthalate (DEHP), di-n-butyl phthalate (DnBP) and butylbenzyl phthalate (BBzP)), and three others were banned for use in toys and childcare articles which children can place in their mouths (di-isononyl phthalate (DiNP), di-isodecyl phthalate (DIDP) and di-n-octyl phthalate (DNOP)) [37].

Certain phthalates, including DEHP, DnBP, BBzP and DnPeP, are prohibited in cosmetics, according to the Cosmetic Products Regulation of 2008 [38].

Under the REACH program, regulations were further established in 2015 for four phthalates (BBzP, DEHP, DnBP and diisobutyl phthalate (DiBP)), prohibiting them from being used in the European Union without authorization. This, however, does not apply to imported goods.

Since June 2017, di-n-pentyl phthalate (DnPeP) has been included on the candidate list of SVHCs that require authorization. Additionally, since 2017, phthalates known to have reprotoxic effects cannot be placed on the EU market as individual substances or in mixtures if they exhibit phthalate levels greater than 0.1% in weight. Additionally, DEHP, DnBP, DiBP, DiNP, DnOP and BBzP are restricted in terms of their use in plasticized materials in children’s articles under the entries 51 and 52 of Annex XVII of REACH. From 2020, DEHP, DnBP, DiBP and BBzP have been further restricted to a concentration equal to or below 0.1% by weight, individually or in any combination, in any plasticized material in articles used by consumers or in indoor areas.

It is important to remark that in the absence of safer materials, companies can opt to obtain authorizations to continue using these phthalates for consumer products.

**Figure 1:**
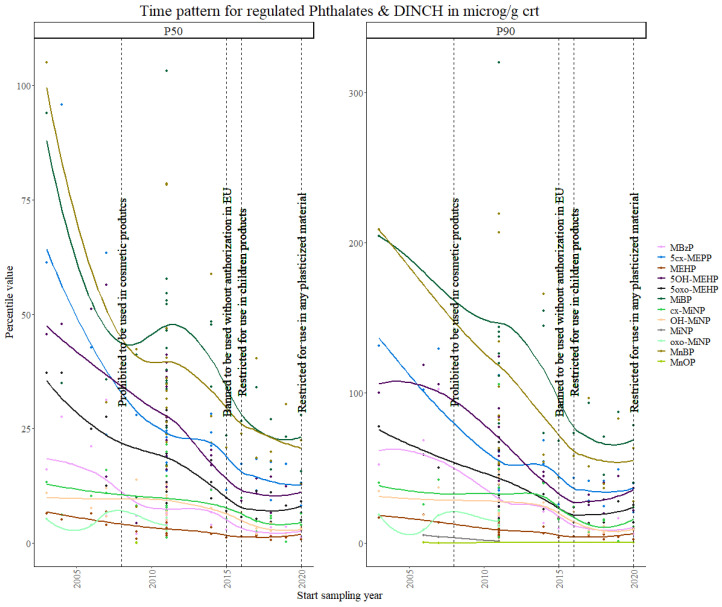
Time patterns for urinary levels of regulated phthalate metabolites in children from 5 to 12 years old. Percentile value is shown in µg/g creatinine.

In general, as it can be seen in Figure 1, internal levels of regulated phthalates follow a clear decreasing trend over time for both the 50th and the 90th percentile, starting even before the earliest regulations on their use, as can be observed in this decrease, independent from the regulations, which is perhaps due to an increasing knowledge by the general population about the usage of plastic consumer products in relation to food and in cosmetic products and manufacturers being aware of upcoming regulations, specifically after the ban of 1999, on their use in children’s toys.

For DEHP metabolites (mono (2-ethylhexyl) phthalate (MEHP), mono (2-ethyl-5-hydroxy-hexyl) phthalate (5OH-MEHP), mono (2-ethyl-5-carboxy-pentyl) phthalate (5cx-MEPP) and mono (2-ethyl-5-oxo-hexyl) phthalate (5oxo-MEHP)), MEHP shows a slight decreasing trend for both the 50th and 90th percentiles, while a significant decrease is observed for the other three. The results from the Theil–Sen regression show significant trends (*p*-value < 0.001) for all the DEHP metabolites.

Mono-n-butyl phthalate (MnBP) and mono-isobutyl phthalate (MiBP) were found to have relatively high concentration values, in comparison with the rest of the regulated phthalate markers, in the children population. They also show the steepest decreasing trend over time for both percentiles. These results are consistent with a German study, where MnBP and MiBP showed the highest levels and the DEHP, DnBP and BBzP metabolite levels declined significantly between the years 2002 and 2008 [39,40]. The results are also in line with a meta-study, which included data from Canada, South Korea and the US, where 5OH-MEHP, 5cx-MPHP and 5oxo-MEHP concentration values decreased more steeply than MEHP in the children population [41], and in the Danish and German adult male population [42]. Flemish studies in teenagers have also shown a significant decrease in the sum of the DEHP metabolites and other phthalate esters and alternative plasticizers from 2003 to 2017 [43].

**Figure 2:**
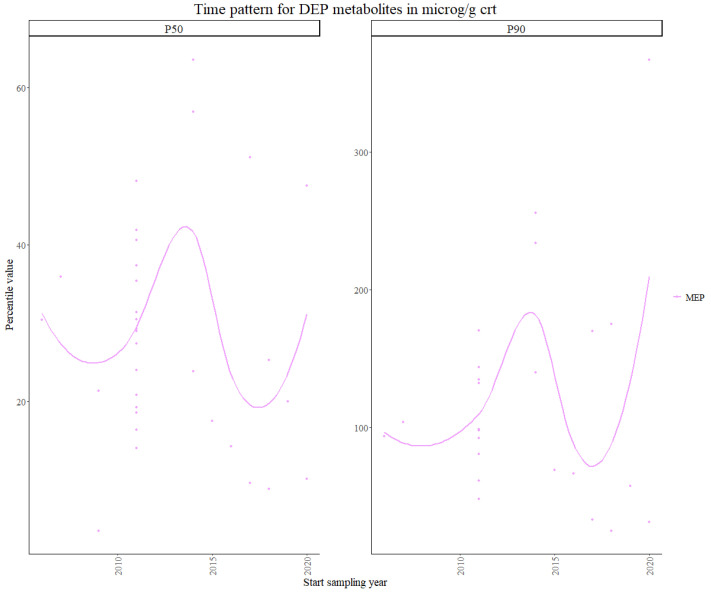
Time patterns for urinary levels of unregulated MEP in children from 5 to 12 years old. Percentile value is shown in µg/g creatinine.

Within the unregulated phthalate metabolites, Figure 2 shows that the highest P50 values were found for mono-ethyl phthalate (MEP), which is known to be used in perfumes or fragrance-containing products such as shampoo, body lotions and other cosmetics [44]. The MEP P50 value for INMA Spain and the MEP P90 value for DEMOCOPHES Spain were excluded from the analysis as they were identified as outliers. The INMA P50 value was around 600 µg/g CRT, while the average P50 value for the rest of the data collection for MEP was around 150 µg/g CRT. For DEMOCOPHES Spain, the P90 value was around 800 µg/g CRT, while the average value for the rest of the data collection was 200 µg/g CRT. The difference in the values caused the trend to be driven by these two values and, therefore, it was decided that removing them resulted in a more realistic trend.

**Figure 3:**
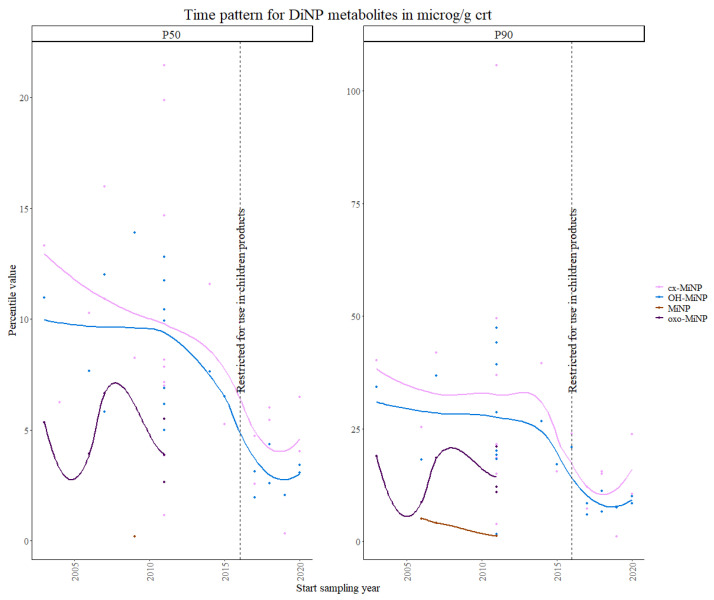
Time patterns for urinary levels of DiNP metabolites in children from 5 to 12 years old. Percentile value is shown in µg/g creatinine.

Moreover, 7-Oxo-(Mono-methyl-octyl) phthalate (Oxo-MiNP) in Figure 3 showed the most variable trend, although percentiles were only available for the first and second time periods.

These data were consistent with the results from the Theil–Sen regression, which showed significant decreasing trends (*p*-values < 0.001) for all the phthalates (regulated and unregulated) included in this study, except for the Oxo-MiNP and mono-n-octyl phthalate (MnOP) (usually one of the least detected biomarkers) for which only enough 90th percentile data were available and a significant increasing trend was found. Oxo-MiNP levels have shown increasing trends in other studies [39], while for MnOP no studies on time trends were found for a comparison of the results.

For DINCH, which is used as a phthalate substitute in product manufacturing, pooled data from 9 studies for the second time point and 12 studies for the third time point, were used in the statistical analysis. No data from the first time point was available in any of the HBM studies identified for this work. There are no current regulations on the use of DINCH.

**Figure 4:**
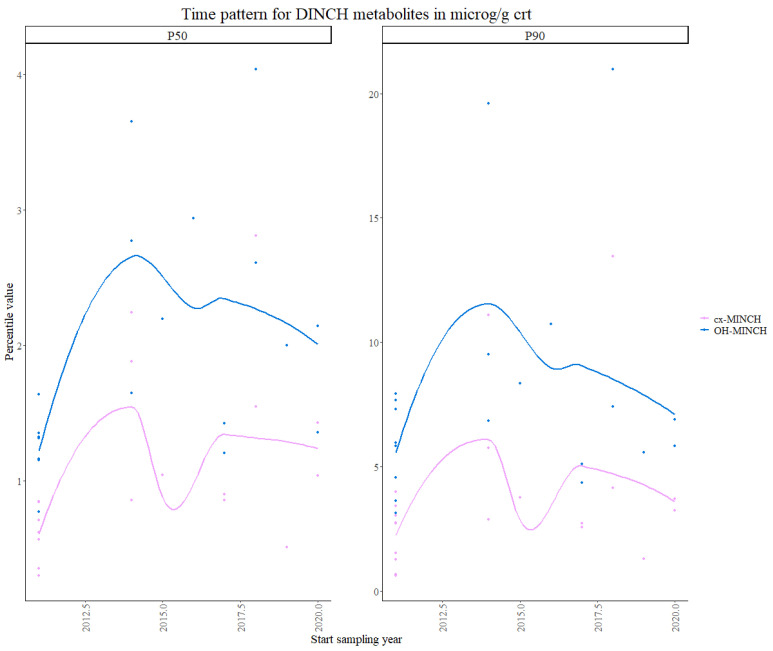
Time patterns for urinary levels of unregulated DINCH metabolites in children from 5 to 12 years old. Percentile value is shown in µg/g creatinine.

Cyclohexane-1,2-dicarboxylate-mono-(7-carboxylate-4-methyl) heptyl ester (cx-MINCH) showed no clear visual overall trend, as seen in Figure 4. The results from the Theil–Sen regression show a significant *p*-value (<0.0001) for the increasing trend over time for both the 50th and 90th percentiles.

Cyclohexane-1,2-dicarboxylate-mono-(7-hydroxy-4-methyl) octyl ester (OH-MINCH) showed a slight increasing trend over time in the visualization of the percentile values, although visually it seemed to decrease after 2017–2018. The results from the Theil–Sen regression showed a significant *p*-value (<0.0001) for the increasing trend over time for both the 50th and 90th percentiles. This is consistent with US studies, in which DINCH showed increasing trends in the US [41], as well as within Germany, where a study following participants from Germany and Denmark between 2000 to 2017 showed increasing trends, although non-significant [42,45].

The results from the Theil–Sen regression for phthalates and DINCH are visualized in Figure S1.

#### 3.1.2. OPFRs

Regarding OPFRs, pooled data over six studies for the second time point and seven studies for the third time point were used for the statistical analysis. No data were available for the first time point in any of the studies used for this work.

**Figure 5:**
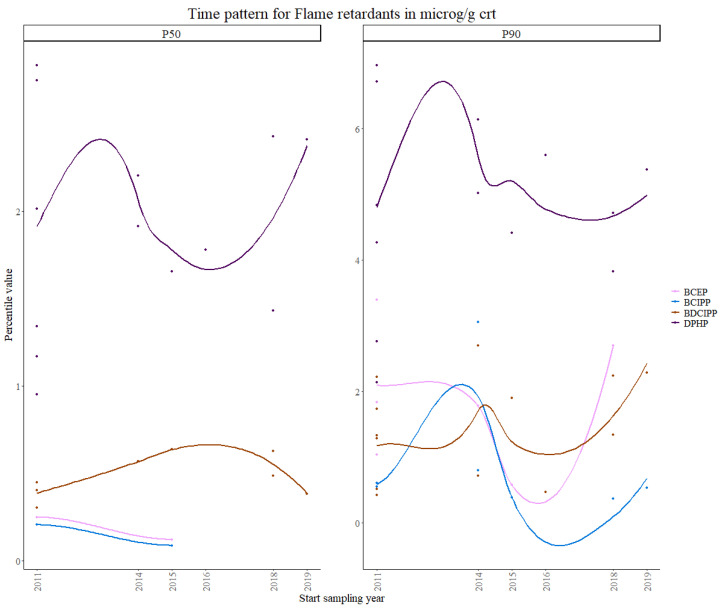
Time patterns for urinary levels of flame retardants in children from 5 to 12 years old. Percentile value is shown in µg/g creatinine.

Urinary biomarkers were available for only four OPFRs (DPHP, BCEP, BCIPP and BDCIPP). No clear trend could be visualized for the overall European population, as observed in Figure 5**.** DPHP showed the highest concentration values in the children population, which is consistent with other US studies [13], while BCEP, BCIPP and BDCIPP showed variable concentration values. For BCEP, a limited set of 50th percentiles were available for only the first and second time periods. Taking this into account, no overall trend can be observed for the 50th percentile, however a decreasing trend was noticeable for the 90th percentile for DPHP, BCIPP and BCEP after 2014, with an increase after 2016 for all the markers. The results from the Theil–Sen regression showed a slightly significant decreasing trend for the 90th percentile of BCEP (*p*-value < 0.03). For BCIPP, a significant decreasing trend was found for both percentiles (*p*-values < 0.001). For BDCIPP, a significant increasing trend was observed (*p*-value < 0.001) for both percentiles, which is consistent with other studies in the US [46] and in Japan [47]. A borderline significant increase was observed for DPHP in the 50th percentile (*p*-value = 0.06), consistent with the increasing European DPHP consumption [48], while a borderline significant decrease was observed in the 90th percentile (*p*-value = 0.05).

There are no current regulations or restrictions on the use of OPFRs in industrial and commercial products in Europe and, to our knowledge, no time trends on OPFRs have been reported. However, it has previously been shown that OPFRs exposure levels are now higher than those of PBDEs, suggesting that concentrations of the former are increasing [49].

The results from the Theil–Sen regression for OPFRs are visualized in Figure S2.

### 3.2. Time Patterns Observed in Adult Women (24–52 Years) in Europe

#### 3.2.1. Cadmium

For urinary cadmium, aggregated data from 13 studies in the first time period, 17 studies in the second time period and 9 studies in the third time period, were used in the statistical analysis.

The maximum cadmium levels in water were provided by the WHO in 2004, namely 3 µg/L and, in 2006, the maximum levels in foodstuffs were revised (Regulation (EC) No. 1881/2006). In 2014, there was a further revision to the maximum tolerated levels in baby foods and chocolate/cocoa products under Commission Regulation (EU) No 488/2014. However, under Commission Regulation (EU) 2021/1323 [50], it was mentioned that the mitigation measures previously implemented do not reduce the presence of cadmium in many foodstuffs, thus encouraging yet another revision to the previous regulations. In addition to regulations, the HBM German Commission has defined HBM I and HBM II health guidance values for cadmium of 0.5 and 2 µg/L, respectively.

**Figure 6:**
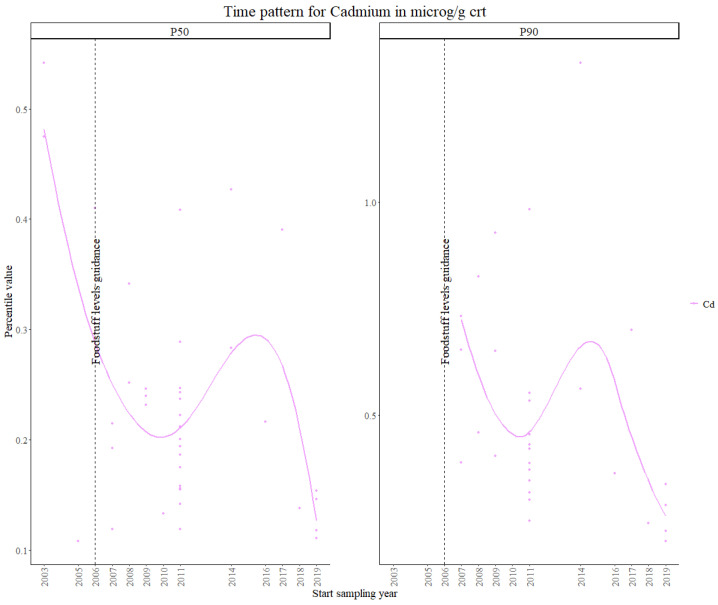
Time patterns for urinary levels of cadmium in women from 24 to 52 years old. Percentile value is shown in µg/g creatinine.

In Figure 6, a decrease in both percentile values within the first time period is observed. From 2010 onwards, the trend increases, followed by yet another decrease after approximately 2015. This suggests that cadmium levels should be further regulated, as human exposure to cadmium in Europe is still fluctuating over time. The results from the Theil–Sen regression show an overall significant decreasing trend for both percentiles.

In the literature, no time trends at the European level have been assessed, however on the country level, cadmium exposure levels in urine have been shown to decrease in the German population; however, the results were not significative [51]^,^ for the Korean population [52] and Belgian teenager population [53]. However, in a study from northern Sweden, exposure values for cadmium in blood between the years 1990 and 2014 remain stable [54].

The results from the Theil–Sen regression for cadmium are visualized in Figure S3.

#### 3.2.2. Bisphenols

For bisphenols, aggregated data from 29 studies were used, i.e., 9 studies in the first time period, 9 studies in the second time period and 11 studies in the third time period.

In the EU, BPA has been banned in infant feeding bottles since June 2011, and in plastic bottles and packaging containing food for babies and children under three years old since September 2018. Furthermore, it has been restricted for use in food contact materials, where its specific migration limit has been reduced from 0.6 mg/kg to 0.05 mg/kg (Commission Regulation (EU) 2018/213 [55]). Additionally, in 2012, the French authorities adopted national law (LOI no. 2012-1442) to ban the use of Bisphenol A in all food contact materials. In 2020, BPA was restricted for use in thermal paper in all EU countries up to 0.02% by weight, following the 2016 REACH Annex XVII addition of BPA to the restricted substances list. However, many companies opted for replacing BPA with BPS in thermal paper. In order to tackle this issue, Germany has proposed a group restriction for BPA and other bisphenols with endocrine-disrupting properties for the environment considering the placing on the market of mixtures and articles where the concentration is equal to or greater than 10 ppm (0.001% by weight).

**Figure 7:**
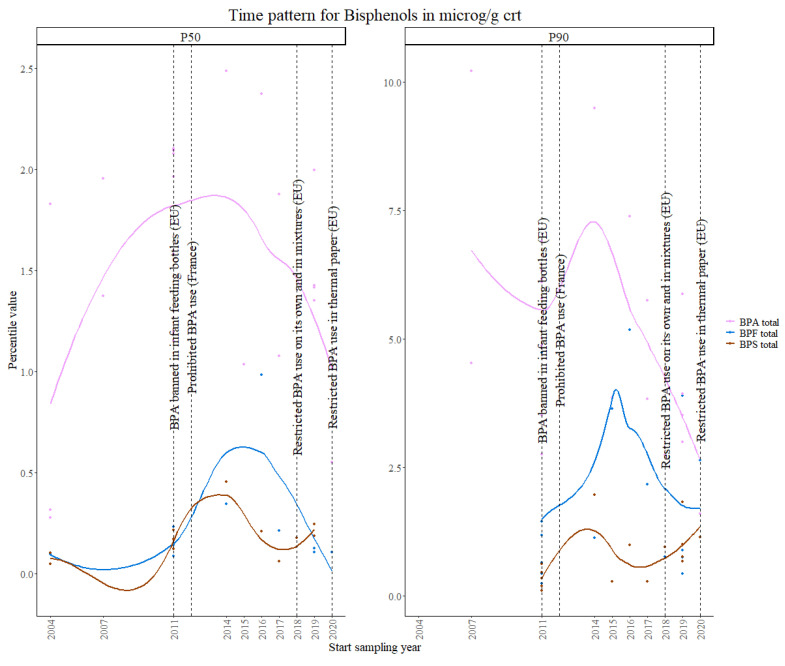
Time patterns for urinary levels of bisphenols in women from 24 to 52 years old. Percentile value is shown in µg/g creatinine.

The timing of the restrictions on the use of bisphenols are indicated in Figure 7, together with the time pattern for BPA urinary concentration levels. An increasing trend can be observed for the 50th percentile data before the prohibition of BPA in the European Union in 2018, specifically until around 2015, followed by a clear decreasing trend. For the 90th percentile, which represents the population with higher exposure levels, a clear decreasing trend in concentration values is shown.

Looking at the most common BPA substitutes, BPS and BPF, an increasing trend is shown for the 50th percentiles for both markers until 2015, after which a clear decrease is observed for BPF, while BPS values show a slight increase. For the 90th percentile, BPF shows the same trend as for the 50th percentile, while BPS has a clear increasing trend. The results from the Theil–Sen regression show a significant decrease in both percentiles for BPA and a significant increase for BPS, therefore suggesting that the prohibitions regarding the use of BPA in commercial products might have resulted in its substitution with BPS. For BPF, a borderline significant increasing trend is observed for the 90th percentile.

Studies from Germany show a non-significant increasing temporal trend in urinary BPA exposure (µg/g creatinine) based on an Environmental Specimen Bank (ESB) study [56], and other studies have shown that BPA intake by adults worldwide decreased until 2008 and increased after 2008 [57]. A study in the US showed a significant decrease in urinary BPA levels from 2010 to 2014, and a slight upward trend for BPS [58].

The results from the Theil–Sen regression for bisphenols are visualized in Figure S4.

#### 3.2.3. PAHs

Finally, for PAHs, aggregated data from five studies in the first time period, six studies in the second time period and ten studies in the third time period, were used in the statistical analysis.

PAHs are regulated based on the National Emission Ceilings Directive 2001/81/EC, and under Commission Regulation (EC) No. 1881/2006 of 19 December 2006, which sets the maximum levels for certain contaminants in foodstuffs. They are also restricted in rubber and in plastic parts for some consumer goods, while eight carcinogenic PAHs are restricted for use in childcare articles and toys, restrictions that were implemented in 2015 [59,60]. Anthracene oil and coal tar pitch are also subject to authorization under REACH. They are also included in the ECHA’s registry of restriction intentions until outcome for 2021.

Several PAHs were analyzed throughout the studies, however, the number of studies that measured the same compounds was limited.

**Figure 8:**
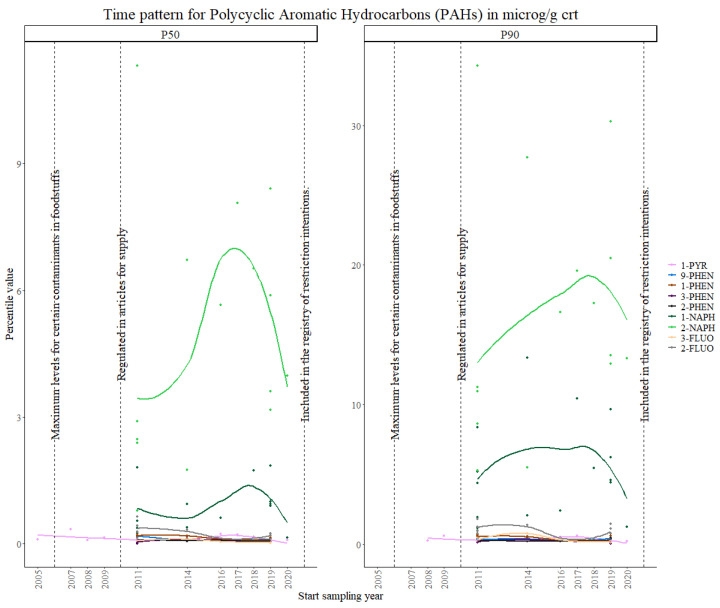
Time patterns for urinary levels of PAHs in women from 24 to 52 years old. Percentile value is shown in µg/g creatinine.

**Figure 9:**
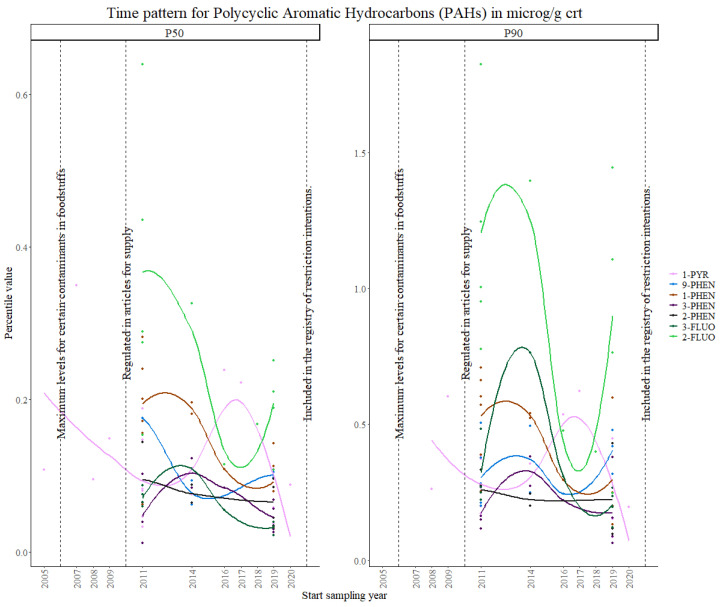
Time patterns for urinary levels of PAHs in women from 24 to 52 years old (without 1-NAPH and 2-NAPH). Percentile value is shown in µg/g creatinine.

Overall, 2-NAPH and 1-NAPH showed the highest exposure levels, as can be seen in Figure 8, consistent with the values from a pooled meta-analysis in children and adolescents [61]. A visual exploration of the time trends (Figure 9), not including 2-NAPH and 1-NAPH, shows highly variable patterns for all the remaining markers, although the overall concentration values seem to decrease.

Overall, 1-PYR was the most interesting case, as enough data points were collected for the three time periods. After the 2005 regulation on the maximum levels in foodstuffs, a decreasing trend in internal concentration levels was observed, followed by an increase with a peak at around 2017, after which levels decrease again. A concave-downward trend is repeated in all other PAHs except for 2-PHEN, which showed the most stable concentration levels over time. The results from the Theil–Sen regression show significant overall decreasing trends in both the P50 and P90 for 1-PYR, 1-PHEN, 2-PHEN and 3-PHEN, and 2-FLUO and 3-FLUO. Significant increasing trends were found for 1-NAPH and 2-NAPH and 9-PHEN, suggesting that stronger regulation should be established.

The German ESB study shows consistent results for 2-NAPH, where increasing exposure values were found between 1995 and 2019, while for 1-NAPH the exposure values decreased significantly. Phenanthrene metabolites also showed a decreasing trend, although for 9-PHE it was not significant. These decreasing trends for the most commonly used PAHs to assess PAH exposure (1-NAPH and 1-PYR) can probably be attributed to smoking bans and regulations [62]. These observations are also in accordance with previous findings from the US National Health and Nutrition Examination Survey (NHANES) in children and adolescents, which showed a correlation between SHS and elevated exposure to 1-PYR and NAPH [63,64].

In 2009, a Council Recommendation was issued on smoke-free environments, which was adopted differently depending on the country. The decrease in PAHs exposure values is probably due to the European effort to adopt and implement laws to fully protect their citizens from tobacco exposure in public places, within 3 years of the Recommendation [65].

The results from the Theil–Sen regression for PAHs are visualized in Figure S5.

#### 3.2.4. Strengths and Limitations

The strongest point of this work is the use of harmonized human biomonitoring data, due to a considerable effort made within the HBM4EU project to cooperate and transfer all the study data into the same format. This has allowed comparable aggregated data from different European regions to be used in this work. Furthermore, this study demonstrates a sustainable use of the available biomonitoring data to answer the question about the effect of regulatory and policy work.

The main limitation to this work lies in the data sets that originate from heterogeneous sources and, therefore, further study design should improve on the harmonization of the data, recruitment, sampling protocols, etc.

Furthermore, to include as many studies as possible, no minimum sample size was established. Correction was made for this in the Theil–Sen regression results in an over estimation of the *p*-values, as addressed in previous sections.

For the first time period, the heterogeneity between the studies was more pronounced as some of the studies’ data was collected from publications. Consequently, some studies for the first time period might include males (in the case of markers measured in adults) or participants outside the age groups selected for this work. Additionally, the algorithms and programs used to calculate the aggregated data obtained from the publications might produce results that differ slightly from the ones used in the harmonization process.

The use of aggregated data has allowed us to include a high number of studies, however, the lack of individual data has resulted in a small number of data points to be used in the analysis for each biomarker, as only the calculated P50 and P90 values were available.

The results from this work make use of cross-sectional studies that were comparable in regard to their target populations. This allows for the use of studies that include participants of the same age group and that are representative of the general population in the catchment area. The use of longitudinal studies to assess the time trends within the same participant group would allow the concentration level changes to be seen over time in exactly the same population. However, it would also limit the ability to determine time trends in the same target population age group. Furthermore, during longitudinal studies, where the same population is followed over time, changes in the individual’s behavior, habits and exposure might happen, making it difficult to assess whether the exposure patterns are due to regulations or due to the aforementioned changes.

The low number of data points, due to working uniquely with the P50 and P90 values, results in a lack of possibility to perform geographical comparisons at the European level. Different regions in Europe differ in regard to cultural habits, diet and even regulations, therefore some trends might be different depending on the region and the substance group investigated. Accordingly, further work is needed to assess regional trends. Additionally, including questionnaire information about the cultural and behavioral differences would allow for a more detailed trend identification between groups.

Finally, biomonitoring data on chemicals, especially for those that are rapidly metabolized, are only snap shots on exposure. Biomarkers measured in a one spot urine sample per participant may not be representative of the exposure of an individual. By including large sample sizes, the biomarker concentration levels better represent the population’s internal exposure level.

## 4. Conclusions

This work collected biomarker levels for phthalates, DINCH and OPFRs in European children (5 to 12 years) and bisphenols, cadmium and PAHs in European women (24 to 52 years). Despite heterogeneity in the data sets that were collected from different countries, time trends could be observed if the data were compared from three time periods: 2000–2010, 2011–2012 and 2014–2021. The resulting time patterns have been compared with the current European regulations to assess whether the effects of implemented policies can be observed in the general population’s exposure levels to specific chemicals. The results from this work show that regulations on some substance groups, such as phthalates or bisphenols, have resulted in the increased levels of substitutes, but that more stringent regulations are needed for the remaining compounds. Specifically, it is shown that regulations on consumer products at the European level are not as effective as they should be, especially for those substances used as a substitute for other biomarkers, perhaps due to a lack of regulation on imported materials and products or the availability of safe substitute materials. Regulated compounds, such as regulated phthalates, showed decreasing concentrations over time. Biomarkers for other chemicals used as substitutes for regulated ones, such as BPS, BPF and DINCH, increased or remained stable over time. Cd and PAHs did not show clear decreasing time trends, and although these compounds are regulated already and have been for a long period, they are still present at levels that cause health concerns and, therefore, they should still be subject to biomonitoring.

As intended by the HBM4EU [66], these results provide input for policy makers to guide their work in terms of which chemicals and substance groups should be further regulated or for which additional measures should be taken.

In conclusion, this work allows changes in the exposure levels for prioritized chemicals in the general population in Europe to be visualized and evaluated. It provides insight on the possible effects that the establishment of regulations might have on human exposure in Europe.

Further work should include research on whether mitigation measurements (REACH restrictions, EFSA restrictions, such as new maximum levels, REACH authorizations, etc.) have been taken at the EU or national level and investigate their effects per region or even per country. Moreover, since new chemicals appear as substitutes for the previous ones, a follow up on the concentrations in the population is crucial to prove the effectiveness of the regulations, as well as to identify new risks to the population.

## Data Availability

All data can be accessed through the European HBM dashboard (https://hbm.vito.be/eu-hbm-dashboard, accessed on 1 August 2023).

## Supplementary Materials

The following supporting information can be downloaded at: https://www.mdpi.com/article/10.3390/toxics11100819/s1, Table S1: Population characteristics of adult women from studies which measured bisphenols; Table S2: Population characteristics for adult women from studies which measured cadmium; Table S3: Population characteristics for adult women from studies which measured PAHs; Table S4: Population characteristics for children from studies which measured phthalates; Table S5: Population characteristics for children from studies which measured DINCH; Table S6: Population characteristics for children from studies which measured OPFRs; Table S7: Analytical technique used to measure all substance groups for all studies in the three time periods: period 1 (2000–2010) includes literature and harmonized aggregated data; period 2 (2011–2012) includes the: DEMOCOPHES studies and period 3 (2014–2022) includes the HBM4EU Aligned studies aggregated data; Figure S1: Forest plots representing the estimate and confidence interval for the Theil-Sen re-gression of phthalates and DINCH measured in children 5–12 years of age for the 50th and 90th percentiles. In black, exposure markers with *p*-value < 0.05 (significant). In grey, exposure markers with *p*-value > 0.05 (unsignificant); Figure S2: Forest plots representing the estimate and confidence interval for the Theil-Sen re-gression of OPFRs measured in children 5–12 years of age for the 50th and 90th percentiles. In black, exposure markers with *p*-value < 0.05 (significant). In grey, exposure markers with *p*-value > 0.05 (unsignificant); Figure S3: Forest plots representing the estimate and confidence interval for the Theil-Sen re-gression of cadmium measured in women 24–52 years of age for the 50th and 90th percentiles. In black, exposure markers with *p*-value < 0.05 (significant). In grey, exposure markers with *p*-value > 0.05 (unsignificant); Figure S4: Forest plots representing the estimate and confidence interval for the Theil-Sen re-gression of bisphenols measured in women 24–52 years of age for the 50th and 90th percentiles. In black, exposure markers with *p*-value < 0.05 (significant). In grey, exposure markers with *p*-value > 0.05 (unsignificant); Figure S5: Forest plots representing the estimate and confidence interval for the Theil-Sen re-gression of PAHs measured in women 24–52 years of age for the 50th and 90th percentiles. In black, exposure markers with *p*-value < 0.05 (significant). In grey, exposure markers with *p*-value > 0.05 (unsignificant).

## Abbreviations

In appearance order:

HBM4EU	European Human Biomonitoring Initiative
HBM	Human biomonitoring
OPFRs	Organophosphorus flame retardants
PAHs	Polycyclic aromatic hydrocarbons
DINCH	1:2-Cyclohexane dicarboxylic acid diisononyl ester
Cd	Cadmium
PFAS	Per- and polyfluoroalkyl substances
FR	Flame retardants
ECHA	European Chemicals Agency
PBDEs	Polybrominated diphenyl ethers
OPFRs	Organophosphate flame retardants
IARC	International Agency for Research on Cancer
BPA	Bisphenol A
BPS	Bisphenol S
BPF	Bisphenol F
SOPs	Standard operating procedures
ICIs	Interlaboratory comparison investigations
EQUAS	External quality assessment schemes
DEHP	Di(2-ethylhexyl) phthalate
DnBP	Di-n-butyl phthalate
BBzP	Butylbenzyl phthalate
DINP	Di-isononyl phthalate
DIDP	Di-isodecyl phthalate
DNOP	Di-n-octyl-phthalate
DIBP	Di-isobutyl phthalate
DnPeP	Di-n-pentyl phthalate
MEHP	Mono(2-ethylhexyl) phthalate
5OH-MEHP	Mono (2-ethyl-5-hydroxy- hexyl) phthalate
5cx-MEPP	Mono (2-ethyl-5-carboxy- pentyl) phthalate
5oxo-MEHP	Mono (2-ethyl-5-oxo-hexyl) phthalate
MEP	Mono-ethyl phthalate
MnBP	Mono-n-butyl phthalate
MiBP	Mono-isobutyl phthalate
Oxo-MiNP	7-Oxo-(Mono-methyl-octyl) phthalate
MnOP	Mono-n-octyl phthalate
cx-MINCH	Cyclohexane-1:2-dicarboxylate-mono-(7-carboxylate-4-methyl) heptyl ester
OH-MINCH	Cyclohexane-1:2-dicarboxylate-mono-(7-hydroxy-4-methyl) octyl ester
ESB	Environmental Specimen Bank
SVHCs	Substance of very high concern
