# Supplementary material for: Time Patterns in Internal Human Exposure Data to Bisphenols, Phthalates, DINCH, Organophosphate Flame Retardants, Cadmium and Polyaromatic Hydrocarbons in Europe
Source: Toxics. 2023 Sep 28;11(10):819. doi: 10.3390/toxics11100819 (PMC10610666; doi:10.3390/toxics11100819)
Supplement: Supplementary file 1 [file toxics-11-00819-s001.zip › toxics-2601066-supplementary.pdf]

toxics

MDPI

Supplementary Materials: Time Patterns in Internal Human Exposure Data to Bisphenols, Phthalates, DINCH, Organophosphate Flame Retardants, Cad-mium and Polyaromatic Hydrocarbons in Europe

Laura Rodriguez Martin, Liese Gilles, Emilie Helte, Agneta Åkesson, Jonas Tägt, Adrian Covaci, An Van Nieuwenhuyse, Andromachi Katsonouri, Amrit K. Sakhi, Elena Anastasi, Anna-Maria Andersson, Beata Janasik, Brice Appenzeller, Catherine Gabriel, Cathrine Thomsen, Darja Mazej, Denis Sarigiannis, Fabio Barbone, Hanna Tolonen, Hanne Frederiksen, Jana Klanova, Jani Koponen, Janja Snoj Tratnik, Koppen Gudrun, Kristin Ólafsdóttir, Liesbeth Knudsen, Loïc Rambaud, Loreta Strumylaite, Lubica Palkovicova Murinova. Lucia Fabelova. Margaux Rioux, Marika Berglund, Maté Szabados, Medea Imboden, Michelle Laeremans, Milada Eštóková, Natasa Janev Holcer, Nicole Probst-Hensch, Nicole Vodrazkova, Nina Vogel, Pavel Piler, Philipp Schmidt, Sónia Namorado, Szilvia Kozepesy, Tamás Szigeti, Thorhallur I Halldorsson, Till Weber, Tina Kold Jensen, Valentina Rosolen, Vladimira Puklova, Ovnair Sepai, Lorraine Stewart, Marike Kolossa, Marta Esteban-López, Argelia Castaño, Jos Bessems, Greet Schoeters and Eva Govarts

Table S1. Population characteristics of adult women from studies which measured bisphenols.

Time Period	Country	Study	Matrix	Total N*	Sampling period	Age range	Reference
Literature data (2000-2010)	ES	INMA	US	120	2004-2008	16-NA	Casas et al. 2011
	NL	Study on Maternal Weight Gain (Early Pregnancy)	US	1213	2004-2005	30.6 (mean)	Philips et al. 2020
	NL	Study on Maternal Weight Gain (Mid-Pregnancy)	US	1213	2004-2005	30.6 (mean)	Philips et al. 2020
	SE	Swedish POPUP Study	US	27	2009	20-41	Gyllenhammar et al. 2017
	SE	Swedish POPUP Study	US	30	2010	20-41	Gyllenhammar et al. 2017
Harmonized aggregated data (2000-2010)	DK	Copenhagen Puberty Sub study 24h	UD	64	2007	12-39	https://ipchem.jrc.ec.europa.eu/RDSIdiscovery/ipchem/index.html#showmetadata/CPHPUB129SUB
	DK	Copenhagen Puberty Sub study day1	UM	64	2007	12-39	https://ipchem.jrc.ec.europa.eu/RDSIdiscovery/ipchem/index.html#showmetadata/CPHPUB129SUB
	DK	Copenhagen Puberty Sub study day2	UM	64	2007	12-39	https://ipchem.jrc.ec.europa.eu/RDSIdiscovery/ipchem/index.html#showmetadata/CPHPUB129SUB
	DE	ESB	UD	90	2007-2009	20-39	https://ipchem.jrc.ec.europa.eu/#showmetadata/ESB
DEMOCOPHES (2011-2012)	BE	DEMOCOPHES	UM	129	2011-2012	27-45	https://ipchem.jrc.ec.europa.eu/RDSIdiscovery/ipchem/index.html#showmetadata/DEMOCOPHESBE
	CZ	DEMOCOPHES	UM	116	2011-2012	29-45	https://ipchem.jrc.ec.europa.eu/RDSIdiscovery/ipchem/index.html#showmetadata/DEMOCOPHESCZ
	DK	DEMOCOPHES	UM	145	2011-2012	31-52	https://ipchem.jrc.ec.europa.eu/RDSIdiscovery/ipchem/index.html#showmetadata/DKDEMOCOPHES
	LU	DEMOCOPHES	UM	60	2011-2012	33-45	https://ipchem.jrc.ec.europa.eu/RDSIdiscovery/ipchem/index.html#showmetadata/DEMOCOPHESLU
	PL	DEMOCOPHES	UM	93	2011-2012	27-46	https://ipchem.jrc.ec.europa.eu/#showmetadata/DEMOCOPHESPL

	SI	DEMOCOPHES	UM	107	2011-2012	30-46	https://ipchem.jrc.ec.europa.eu/RDSIdiscovery/ipchem/index.html#showmetadata/SLODEMOCOPHES
	ES	DEMOCOPHES	UM	117	2011-2012	26-48	https://ipchem.jrc.ec.europa.eu/RDSIdiscovery/ipchem/index.html#showmetadata/DEMOCOPHESSPAIN
	CY	DEMOCOPHES	UM	60	2011-2012	27-45	https://ipchem.jrc.ec.europa.eu/index.html#showmetadata/DEMOCOPHESCY
	SE	DEMOCOPHES	UM	98	2011-2012	28-46	https://ipchem.jrc.ec.europa.eu/RDSIdiscovery/ipchem/index.html#showmetadata/DEMOCOPHESSE
HBM4EU Aligned Studies (2014-2021)	CZ	CELSPAC: YA	UM	148	2019	20-31	https://ipchem.jrc.ec.europa.eu/#showmetadata/CELSPACYA
	DK	CPHMINIPUB_DYMS	US	118	2018-2019	25-39	https://ipchem.jrc.ec.europa.eu/index.html#showmetadata/DYMS
	IS	Diet_HBM	US	114	2019-2021	20-39	https://ipchem.jrc.ec.europa.eu/#showmetadata/DIETHBM
	DE	ESB	UD	90	2014-2021	20-29	https://ipchem.jrc.ec.europa.eu/index.html#showmetadata/ESB
	FR	ESTEBAN	UM	81	2014-2016	20-39	https://ipchem.jrc.ec.europa.eu/#showmetadata/ESTEBAN
	FI	FinHealth	US	157	2017	25-39	https://ipchem.jrc.ec.europa.eu/#showmetadata/FINHEALTH
	HR	HBM survey in adults in Croatia	UM	159	2019-2020	20-39	https://ipchem.jrc.ec.europa.eu/index.html#showmetadata/HBMSURVEYINADULTSINCROATIA
	CH	HBM4EU-study Switzerland	UM	138	2020	21-39	https://ipchem.jrc.ec.europa.eu/#showmetadata/HBM4EUSTUDYINSWITZERLAND
	PT	INSEF-ExpoQuim	UM	171	2019-2020	28-39	https://ipchem.jrc.ec.europa.eu/#showmetadata/INSEFEXPOQUIM
	LU	Oriscav-Lux2	US	111	2016-2018	25-39	https://ipchem.jrc.ec.europa.eu/#showmetadata/ORISCAVLUX2
	PL	POLAES	US	158	2017	21-39	https://ipchem.jrc.ec.europa.eu/#showmetadata/POLAES

UD = Urine 24 hours, US = Urine random spot, UM = First morning urine.

* In case more than one biomarker is measured, the maximum observations per biomarker have been taken.

Table S2. Population characteristics for adult women from studies which measured cadmium.

Time Period	Country	Study	Matrix	Total N**	Sampling period	age range	reference
Literature data (2000-2010)	FR	ENNS	US	1206	2006-2007	18-74	Bechaux et al. 2014
	FR	IMEPOGE	UM	968	2008-2010	20-59	Nisse et al. 2017
	ES	Ria of Huelva (Huelva)*	US	857	2003-2004	18-69	Aguiliera et al. 2010
	ES	Ria of Huelva (Other cities)*	US	861	2003-2004	18-69	Aguiliera et al. 2010
	IT	Modena Solid Waste Incinerator*	US	103	2010	48 (mean)	Ranzi et al. 2013
	ES	ISCIII Biomonitoring Study*	UM	45	2007-2010	25-45	Sánchez-Rodríguez et al. 2015
	DE	Biomonitoring Trace Elements*	UM	87	2005	18-65	Heitland et al. 2006

Harmonized aggregated data (2000-2010)	SI	SLO-HBM-I	US	61	2009	20-39	https://ipchem.jrc.ec.europa.eu/RDSIdiscovery/ipchem/index.html#showmetadata/SLOHBM
	ES	BIOAMBIENT.ES	UM	907	2009-2010	12-59	https://ipchem.jrc.ec.europa.eu/RDSIdiscovery/ipchem/index.html#showmetadata/BIOAMBIENTES
	LT	Breast Cancer Study-1-LT	UM	107	2007-2008	12-59	https://ipchem.jrc.ec.europa.eu/RDSIdiscovery/ipchem/index.html#showmetadata/BCAS1
	CZ	CzechHBM-AE_2007	US	94	2007	20-39	https://ipchem.jrc.ec.europa.eu/RDSIdiscovery/ipchem/index.html#showmetadata/CZECHHBMAE2007
	CZ	CzechHBM-AE_2009	US	98	2009	20-39	https://ipchem.jrc.ec.europa.eu/index.html#showmetadata/CZECHHBMAE2009
	BE	FLEHS 2 adults	UM	98	2008-2009	20-39	https://ipchem.jrc.ec.europa.eu/RDSIdiscovery/ipchem/index.html#showmetadata/FLEHS2REFADULT
DEMOCOPHES (2011-2012)	ES	DEMOCOPHES	UM	120	2011-2012	26-48	https://ipchem.jrc.ec.europa.eu/RDSIdiscovery/ipchem/index.html#showmetadata/DEMOCOPHESSPAIN
	CY	DEMOCOPHES	UM	60	2011-2012	27-45	https://ipchem.jrc.ec.europa.eu/index.html#showmetadata/DEMOCOPHESCY
	CH	DEMOCOPHES	UM	117	2011-2012	27-45	https://ipchem.jrc.ec.europa.eu/#showmetadata/DEMOCOPHES
	IE	DEMOCOPHES	UM	120	2011-2012	25-46	https://ipchem.jrc.ec.europa.eu/#showmetadata/DEMOCOPHES
	PT	DEMOCOPHES	UM	117	2011-2012	26-45	https://ipchem.jrc.ec.europa.eu/#showmetadata/DEMOCOPHES
	RO	DEMOCOPHES	UM	117	2011-2012	25-45	https://ipchem.jrc.ec.europa.eu/#showmetadata/DEMOCOPHES
	UK	DEMOCOPHES	UM	21	2011-2012	32-50	https://ipchem.jrc.ec.europa.eu/#showmetadata/DEMOCOPHES
	SI	DEMOCOPHES	UM	120	2011-2012	30-46	https://ipchem.jrc.ec.europa.eu/RDSIdiscovery/ipchem/index.html#showmetadata/SLODEMOCOPHES
	SE	DEMOCOPHES	UM	100	2011-2012	28-46	https://ipchem.jrc.ec.europa.eu/RDSIdiscovery/ipchem/index.html#showmetadata/DEMOCOPHESSE
	LU	DEMOCOPHES	UM	60	2011-2012	33-45	https://ipchem.jrc.ec.europa.eu/RDSIdiscovery/ipchem/index.html#showmetadata/DEMOCOPHESLU
	PL	DEMOCOPHES	UM	120	2011-2012	27-46	https://ipchem.jrc.ec.europa.eu/#showmetadata/DEMOCOPHESPL
	CZ	DEMOCOPHES	UM	120	2011-2012	29-45	https://ipchem.jrc.ec.europa.eu/RDSIdiscovery/ipchem/index.html#showmetadata/DEMOCOPHESCZ
	HU	DEMOCOPHES	UM	120	2011-2012	26-45	https://ipchem.jrc.ec.europa.eu/RDSIdiscovery/ipchem/index.html#showmetadata/DEMOCOPHESHU
	DE	DEMOCOPHES	UM	112	2011	29-43	https://ipchem.jrc.ec.europa.eu/RDSIdiscovery/ipchem/index.html#showmetadata/DEMOCOPHESDE
	DK	DEMOCOPHES	UM	144	2011-2012	31-52	https://ipchem.jrc.ec.europa.eu/RDSIdiscovery/ipchem/index.html#showmetadata/DKDEMOCOPHES
	SK	DEMOCOPHES	UM	129	2011-2012	24-46	https://ipchem.jrc.ec.europa.eu/#showmetadata/DEMOCOPHESSK
	BE	DEMOCOPHES	UM	129	2011-2012	27-45	https://ipchem.jrc.ec.europa.eu/RDSIdiscovery/ipchem/index.html#showmetadata/DEMOCOPHESBE
HBM4EU Aligned Studies (2014-2021)	CZ	CELSPAC: YA	UM	155	2019	20-31	https://ipchem.jrc.ec.europa.eu/#showmetadata/CELSPACYA
	DK	CPHMINIPUB_DYMS	US	122	2018-2019	25-39	https://ipchem.jrc.ec.europa.eu/index.html#showmetadata/DYMS
	IS	Diet_HBM	US	114	2019-2021	20-39	https://ipchem.jrc.ec.europa.eu/#showmetadata/DIETHBM
	DE	ESB	UD	144	2014-2021	20-29	https://ipchem.jrc.ec.europa.eu/index.html#showmetadata/ESB
	FR	ESTEBAN	UM	216	2014-2016	20-39	https://ipchem.jrc.ec.europa.eu/#showmetadata/ESTEBAN

HR	HBM survey in adults in Croatia	UM	159	2019-2020	20-39	https://ipchem.jrc.ec.europa.eu/index.html#showmetadata/HBMSURVEYINADULTSINCROATIA
PT	INSEF-ExpoQuim	UM	171	2019-2020	28-39	https://ipchem.jrc.ec.europa.eu/#showmetadata/INSEFEXPOQUIM
LU	Oriscav-Lux2	US	111	2016-2018	25-39	https://ipchem.jrc.ec.europa.eu/#showmetadata/ORISCAVLUX2
PL	POLAES	US	158	2017	21-39	https://ipchem.jrc.ec.europa.eu/#showmetadata/POLAES

UD = Urine 24 hours, US = Urine random spot, UM = First morning urine.

* Includes male and female participants.

** In case more than one biomarker is measured, the maximum observations per biomarker have been taken.

Table S3. Population characteristics for adult women from studies which measured PAHs.

Time Period	Country	Study	Matrix	Total N**	Sampling period	Age range	Reference
Literature data (2000-2010)	UK	MATCH*	UM	85	2005-2007	18 - 66	Aquilina et al. 2010
	ES	INMA	UM	204	2003-2005	16 - 36	Llop et al. 2008
	PL	REPRO_PL	US	104	2007-2011	20 - 40	Polanska et al. 2014
Harmonized aggregated data (2000-2010)	ES	BIOAMBIENT.ES	UM	450	2009-2010	12-59	https://ipchem.jrc.ec.europa.eu/RDSIdiscovery/ipchem/index.html#showmetadata/BIOAMBIENTES
	BE	FLEHS 2 adults	UM	448	2008-2009	20-39	https://ipchem.jrc.ec.europa.eu/RDSIdiscovery/ipchem/index.html#showmetadata/FLEHS2REFADULT
DEMOCOPHES (2011-2012)	CZ	DEMOCOPHES	UM	116	2011-2012	29-45	https://ipchem.jrc.ec.europa.eu/RDSIdiscovery/ipchem/index.html#showmetadata/DEMOCOPHESCZ
	DE	DEMOCOPHES	UM	114	2011	29-43	https://ipchem.jrc.ec.europa.eu/RDSIdiscovery/ipchem/index.html#showmetadata/DEMOCOPHESDE
	LU	DEMOCOPHES	UM	59	2011-2012	33-45	https://ipchem.jrc.ec.europa.eu/RDSIdiscovery/ipchem/index.html#showmetadata/DEMOCOPHESLU
	PL	DEMOCOPHES	UM	93	2011-2012	27-46	https://ipchem.jrc.ec.europa.eu/#showmetadata/DEMOCOPHESPL
	CY	DEMOCOPHES	UM	60	2011-2012	27-45	https://ipchem.jrc.ec.europa.eu/index.html#showmetadata/DEMOCOPHESCY
	SE	DEMOCOPHES	UM	94	2011-2012	28-46	https://ipchem.jrc.ec.europa.eu/RDSIdiscovery/ipchem/index.html#showmetadata/DEMOCOPHESSE
HBM4EU Aligned Studies (2014-2021)	CZ	CELSPAC: YA	UM	155	2019	20-31	https://ipchem.jrc.ec.europa.eu/#showmetadata/CELSPACYA
	DK	CPHMINIPUB_DYMS	US	97	2018-2019	25-39	https://ipchem.jrc.ec.europa.eu/index.html#showmetadata/DYMS
	IS	Diet_HBM	US	114	2019-2021	20-39	https://ipchem.jrc.ec.europa.eu/#showmetadata/DIETHBM
	DE	ESB	UD	164	2014-2021	20-29	https://ipchem.jrc.ec.europa.eu/index.html#showmetadata/ESB
	FR	ESTEBAN	UM	120	2014-2016	20-39	https://ipchem.jrc.ec.europa.eu/#showmetadata/ESTEBAN
	HR	HBM survey in adults in Croatia	UM	159	2019-2020	20-39	https://ipchem.jrc.ec.europa.eu/index.html#showmetadata/HBMSURVEYINADULTSINCROATIA
	CH	HBM4EU-study Switzerland	UM	138	2020	21-39	https://ipchem.jrc.ec.europa.eu/#showmetadata/HBM4EUSTUDYINSWITZERLAND
	PT	INSEF-ExpoQuim	UM	171	2019-2020	28-39	https://ipchem.jrc.ec.europa.eu/#showmetadata/INSEFEXPOQUIM
	LU	Oriscav-Lux2	US	111	2016-2018	25-39	https://ipchem.jrc.ec.europa.eu/#showmetadata/ORISCAVLUX2

	PL	POLAES	US	156	2017	21-39	https://ipchem.jrc.ec.europa.eu/#showmetadata/POLAES

UD = Urine 24 hours, US = Urine random spot, UM = First morning urine.

** In case more than one biomarker is measured, the maximum observations per biomarker have been taken.

Table S4. Population characteristics for children from studies which measured phthalates.

Time Period	Country	Study	Matrix	Total N**	Sampling period	Age range	Reference
Literature data (2000-2010)	DK	Childhood Exposure to Phthalates*	US	991	2006-2007	4-9	Boas et al. 2010
	AT	Consumer Habits and phthalate exposure	UM	44	2009	8	Wallner et al. 2016
	DE	Duisburg.Bochum.DE	UM	465	2009-2010	7.8-10.8	Kaspersen-Sonnenberg et al. 2014
	DE	German children exposure to phthalates	US	111	2007	5-6	Koch et al. 2011
	ES	INMA	US	19	2004-2008	4	Casas et al. 2011
Harmonized aggregated data (2000-2010)	DK	Copenhagen Mother-Child cohort	US	659	2006-2007	6-11	https://ipchem.jrc.ec.europa.eu/RDSIdiscovery/ipchem/index.html#showmetadata/CPHMC
	DK	Copenhagen Puberty Study	UM	913	2006-2008	6-11	https://ipchem.jrc.ec.europa.eu/RDSIdiscovery/ipchem/index.html#showmetadata/CPHPUBCROSS
	DK	Copenhagen Puberty Substudy_24h	UD	65	2007	6-11	https://ipchem.jrc.ec.europa.eu/RDSIdiscovery/ipchem/index.html#showmetadata/CPHPUB129SUB
	DK	Copenhagen Puberty Substudy_day 1	UM	65	2007	6-11	https://ipchem.jrc.ec.europa.eu/RDSIdiscovery/ipchem/index.html#showmetadata/CPHPUB129SUB
	DK	Copenhagen Puberty Substudy_day 2	UM	65	2007	6-11	https://ipchem.jrc.ec.europa.eu/RDSIdiscovery/ipchem/index.html#showmetadata/CPHPUB129SUB
	DE	GerES IV (unweighted)	UM	301	2003-2006	6-11	https://ipchem.jrc.ec.europa.eu/#showmetadata/GERESIV
DEMOCOPHES (2011-2012)	BE	DEMOCOPHES	UM	129	2011-2012	6-11	https://ipchem.jrc.ec.europa.eu/RDSIdiscovery/ipchem/index.html#showmetadata/DEMOCOPHESBE
	CZ	DEMOCOPHES	UM	120	2011-2012	6-11	https://ipchem.jrc.ec.europa.eu/RDSIdiscovery/ipchem/index.html#showmetadata/DEMOCOPHESCZ
	CH	DEMOCOPHES	UM	119	2011-2012	6-11	https://ipchem.jrc.ec.europa.eu/#showmetadata/DEMOCOPHES
	IE	DEMOCOPHES	UM	120	2011-2012	6-11	https://ipchem.jrc.ec.europa.eu/#showmetadata/DEMOCOPHES
	PT	DEMOCOPHES	UM	116	2011-2012	5-11	https://ipchem.jrc.ec.europa.eu/#showmetadata/DEMOCOPHES
	RO	DEMOCOPHES	UM	119	2011-2012	6-11	https://ipchem.jrc.ec.europa.eu/#showmetadata/DEMOCOPHES
	UK	DEMOCOPHES	UM	21	2011-2012	6-11	https://ipchem.jrc.ec.europa.eu/#showmetadata/DEMOCOPHES
	DK	DEMOCOPHES	UM	143	2011-2012	6-11	https://ipchem.jrc.ec.europa.eu/RDSIdiscovery/ipchem/index.html#showmetadata/DKDEMOCOPHES

	DE	DEMOCOPHES	UM	119	2012	5-11	https://ipchem.jrc.ec.europa.eu/RDSIdiscovery/ipchem/index.html#showmetadata/DEMOCOPHESDE
	HU	DEMOCOPHES	UM	120	2011-2012	6-11	https://ipchem.jrc.ec.europa.eu/RDSIdiscovery/ipchem/index.html#showmetadata/DEMOCOPHESHU
	LU	DEMOCOPHES	UM	60	2011-2012	6-11	https://ipchem.jrc.ec.europa.eu/RDSIdiscovery/ipchem/index.html#showmetadata/DEMOCOPHESLU
	PL	DEMOCOPHES	UM	120	2011-2012	6-11	https://ipchem.jrc.ec.europa.eu/#showmetadata/DEMOCOPHESPL
	SK	DEMOCOPHES	UM	120	2011-2012	6-11	https://ipchem.jrc.ec.europa.eu/#showmetadata/DEMOCOPHESSK
	SI	DEMOCOPHES	UM	119	2011-2012	6-12	https://ipchem.jrc.ec.europa.eu/RDSIdiscovery/ipchem/index.html#showmetadata/SLODEMOCOPHES
	ES	DEMOCOPHES	UM	120	2011-2012	6-11	https://ipchem.jrc.ec.europa.eu/RDSIdiscovery/ipchem/index.html#showmetadata/DEMOCOPHESSPAIN
	CY	DEMOCOPHES	UM	60	2011-2012	6-11	https://ipchem.jrc.ec.europa.eu/index.html#showmetadata/DEMOCOPHESCY
	SE	DEMOCOPHES	UM	98	2011-2012	6-11	https://ipchem.jrc.ec.europa.eu/RDSIdiscovery/ipchem/index.html#showmetadata/DEMOCOPHESSE
HBM4EU Aligned Studies (2014-2021)	BE	3xG	UM	133	2019-2020	6-8	https://ipchem.jrc.ec.europa.eu/index.html#showmetadata/3XG
	EL	CROME	UM	161	2020-2021	6-11	https://ipchem.jrc.ec.europa.eu/#showmetadata/CROME
	FR	ESTEBAN	UM	286	2014-2016	6-12	https://ipchem.jrc.ec.europa.eu/#showmetadata/ESTEBAN
	DE	GerES V-sub (unweighted)	UM	300	2015-2017	6-12	https://ipchem.jrc.ec.europa.eu/index.html#showmetadata/GERESV
	HU	InAirQ	US	262	2017-2018	8-11	https://ipchem.jrc.ec.europa.eu/index.html#showmetadata/INAIRQ
	IT	NACII	US	299	2014-2016	6-8	https://ipchem.jrc.ec.europa.eu/index.html#showmetadata/NACII
	NO	NEBII	US	300	2016-2017	7-11	https://ipchem.jrc.ec.europa.eu/#showmetadata/NEBII
	DK	OCC	US	300	2018-2019	6-7	https://ipchem.jrc.ec.europa.eu/index.html#showmetadata/OCC
	SK	PCB cohort	US	300	2014-2017	10-12	https://ipchem.jrc.ec.europa.eu/index.html#showmetadata/PCBCOHORT
	PL	POLAES	US	300	2017	7-10	https://ipchem.jrc.ec.europa.eu/#showmetadata/POLAES
	SI	SLO CRP	UM	149	2018	7-10	https://ipchem.jrc.ec.europa.eu/index.html#showmetadata/SLOCRP
	NL	SPECIMEn-NL	US	89	2020	6-11	https://ipchem.jrc.ec.europa.eu/#showmetadata/SPECIMENNL

UD = Urine 24 hours, US = Urine random spot, UM = First morning urine.

* Only sex-stratified data.

** In case more than one biomarker is measured, the maximum observations per biomarker have been taken.

Table S5. Population characteristics for children from studies which measured DINCH.

Time Period	Country	Study	Matrix	Total N**	Sampling period	Age range	Reference
DEMOCOPHES (2011-2012)	BE	DEMOCOPHES	UM	117	2011-2012	6-11	https://ipchem.jrc.ec.europa.eu/RDSIdiscovery/ipchem/index.html#showmetadata/DEMOCOPHESBE
	CZ	DEMOCOPHES	UM	118	2011-2012	6-11	https://ipchem.jrc.ec.europa.eu/RDSIdiscovery/ipchem/index.html#showmetadata/DEMOCOPHESCZ
	DK	DEMOCOPHES	UM	143	2011-2012	6-11	https://ipchem.jrc.ec.europa.eu/RDSIdiscovery/ipchem/index.html#showmetadata/DKDEMOCOPHES

	DE	DEMOCOPHES	UM	112	2011	5-11	https://ipchem.jrc.ec.europa.eu/RDSIdiscovery/ipchem/index.html#showmetadata/DEMOCOPHESDE
	LU	DEMOCOPHES	UM	59	2011-2012	6-11	https://ipchem.jrc.ec.europa.eu/RDSIdiscovery/ipchem/index.html#showmetadata/DEMOCOPHESLU
	PL	DEMOCOPHES	UM	94	2011-2012	6-11	https://ipchem.jrc.ec.europa.eu/#showmetadata/DEMOCOPHESPL
	ES	DEMOCOPHES	UM	120	2011-2012	6-11	https://ipchem.jrc.ec.europa.eu/RDSIdiscovery/ipchem/index.html#showmetadata/DEMOCOPHESSPAIN
	CY	DEMOCOPHES	UM	60	2011-2012	6-11	https://ipchem.jrc.ec.europa.eu/index.html#showmetadata/DEMOCOPHESCY
	SE	DEMOCOPHES	UM	99	2011-2012	6-11	https://ipchem.jrc.ec.europa.eu/RDSIdiscovery/ipchem/index.html#showmetadata/DEMOCOPHESSE
HBM4EU Aligned Studies (2014-2021)	BE	3xG	UM	133	2019-2020	6-8	https://ipchem.jrc.ec.europa.eu/index.html#showmetadata/3XG
	EL	CROME	UM	161	2020-2021	6-11	https://ipchem.jrc.ec.europa.eu/#showmetadata/CROME
	FR	ESTEBAN	UM	286	2014-2016	6-12	https://ipchem.jrc.ec.europa.eu/#showmetadata/ESTEBAN
	DE	GerES V-sub (unweighted)	UM	299	2015-2017	6-12	https://ipchem.jrc.ec.europa.eu/index.html#showmetadata/GERESV
	HU	InAirQ	US	262	2017-2018	8-11	https://ipchem.jrc.ec.europa.eu/index.html#showmetadata/INAIRQ
	IT	NACII	US	300	2014-2016	6-8	https://ipchem.jrc.ec.europa.eu/index.html#showmetadata/NACII
	NO	NEBII	US	300	2016-2017	7-11	https://ipchem.jrc.ec.europa.eu/#showmetadata/NEBII
	DK	OCC	US	300	2018-2019	6-7	https://ipchem.jrc.ec.europa.eu/index.html#showmetadata/OCC
	SK	PCB cohort	US	300	2014-2017	10-12	https://ipchem.jrc.ec.europa.eu/index.html#showmetadata/PCBCOHORT
	PL	POLAES	US	300	2017	7-10	https://ipchem.jrc.ec.europa.eu/#showmetadata/POLAES
	SI	SLO CRP	UM	149	2018	7-10	https://ipchem.jrc.ec.europa.eu/index.html#showmetadata/SLOCRP
	NL	SPECIMEn-NL	US	89	2020	6-11	https://ipchem.jrc.ec.europa.eu/#showmetadata/SPECIMENNL

UD = Urine 24 hours, US = Urine random spot, UM = First morning urine.

** In case more than one biomarker is measured, the maximum observations per biomarker have been taken.

Table S6. Population characteristics for children from studies which measured OPFRs.

Time Period	Country	Study	Matrix	Total N**	Sampling period	Age range	Reference
DEMOCOPHES (2011-2012)	BE	DEMOCOPHES	UM	117	2011-2012	6-11	https://ipchem.jrc.ec.europa.eu/RDSIdiscovery/ipchem/index.html#showmetadata/DEMOCOPHESBE
	CZ	DEMOCOPHES	UM	116	2011-2012	6-11	https://ipchem.jrc.ec.europa.eu/RDSIdiscovery/ipchem/index.html#showmetadata/DEMOCOPHESCZ
	DE	DEMOCOPHES	UM	111	2011	5-11	https://ipchem.jrc.ec.europa.eu/RDSIdiscovery/ipchem/index.html#showmetadata/DEMOCOPHESDE
	SI	DEMOCOPHES	UM	114	2011-2012	6-12	https://ipchem.jrc.ec.europa.eu/RDSIdiscovery/ipchem/index.html#showmetadata/SLODEMOCOPHES
	CY	DEMOCOPHES	UM	60	2011-2012	6-11	https://ipchem.jrc.ec.europa.eu/index.html#showmetadata/DEMOCOPHESCY
	SE	DEMOCOPHES	UM	99	2011-2012	6-11	https://ipchem.jrc.ec.europa.eu/RDSIdiscovery/ipchem/index.html#showmetadata/DEMOCOPHESSE
HBM4EU Aligned Studies (2014-2021)	BE	3xG	UM	133	2019-2020	6-8	https://ipchem.jrc.ec.europa.eu/index.html#showmetadata/3XG
	FR	ESTEBAN	UM	299	2014-2016	6-12	https://ipchem.jrc.ec.europa.eu/#showmetadata/ESTEBAN
	DE	GerES V-sub (unweighted)	UM	300	2015-2017	6-12	https://ipchem.jrc.ec.europa.eu/index.html#showmetadata/GERESV
	NO	NEBII	US	300	2016-2017	7-11	https://ipchem.jrc.ec.europa.eu/#showmetadata/NEBII
	DK	OCC	US	291	2018-2019	6-7	https://ipchem.jrc.ec.europa.eu/index.html#showmetadata/OCC
	SK	PCB cohort	US	300	2014-2017	10-12	https://ipchem.jrc.ec.europa.eu/index.html#showmetadata/PCBCOHORT
	SL	SLO CRP	UM	147	2018	7-10	https://ipchem.jrc.ec.europa.eu/index.html#showmetadata/SLOCRP

UD = Urine 24 hours, US = Urine random spot, UM = First morning urine.

** In case more than one biomarker is measured, the maximum observations per biomarker have been taken.

Table S7. Analytical technique used to measure all substance groups for all studies in the three time periods: period 1 (2000-2010) includes literature and harmonized aggregated data; period 2 (2011-2012) includes the: DEMOCOPHES studies and period 3 (2014-2022) includes the HBM4EU Aligned studies aggregated data.

Time Point	Country	Study name	Substance group	Specific biomarkers	Technique
1 - Literature data	Sweden	Swedish POPUP Study	Bisphenols		LC-MS/MS
	The Netherlands	Study on Maternal Weight Gain			HPLC-ESI-MS/MS
	Spain	INMA			HPLC-MS/MS
	France	IMEPOGE	Cadmium		ICP-MS
	France	ENNS			ICP-MS
	Germany	Biomonitoring Trace Elements			ICP-MS
	Spain	Ria of Huelva			AAS
	Spain	ISCIII Biomonitoring Study			DRC-ICP-MS
	Italy	Modena Solid Waste Incinerator			ICP-MS
	Denmark	Childhood Exposure to Phthalates	Phthalates		LC-MS/MS
	Austria	Consumer Habits and phthalate exposure			LC-ESI-MS/MS
	Germany	Duisburg.Bochum.DE / German children exposure to phthalates			LC/LC–MS/MS

	Spain	INMA			HPLC-MS/MS
	Spain	INMA	Polycyclic Aromatic Hydrocarbons (PAHs)		HPLC-FD
	United Kingdom	MATCH			LC-MS/MS
	Poland	REPRO_PL			GC-MS
1 - Harmonized aggregated data	Denmark	Copenhagen Mother-Child cohort	Phthalates		LC-MS/MS
	Denmark	Copenhagen Puberty Study			LC-MS/MS
	Denmark	Copenhagen Puberty SubStudy (Day1, Day2, 24 hours)			LC-MS/MS
	Germany	GerES IV			LC–MS/MS
	Spain	BIOAMBIENT.ES	Polycyclic Aromatic Hydrocarbons (PAHs)		HPLC-FD
	Belgium	FLEHS 2 Adults			HPLC - FD
	Slovenia	SLO-HBM-I	Cadmium		ICP-MS
	Spain	BIOAMBIENT.ES			ICP-MS
	Lithuania	Breast Cancer Study-1-LT			AAS
	Czech Republic	CzechHBM-AE_2007			Not specified
	Czech Republic	CzechHBM-AE_2009			Not specified
	Belgium	FLEHS 2 Adults			ICP-DRC-MS
	Denmark	Copenhagen Puberty SubStudy (Day1, Day2, 24 hours)	Bisphenols		LC-MS/MS
	Germany	ESB-DE			HPLC–MS/MS
2- DEMOCOPHES	Spain	DEMOCOPHES - ES	Bisphenols	BPA	LC–MS
	Spain	DEMOCOPHES - ES		BPS, BPF	On-line SPE LC-MS/MS
	Czech Republic	DEMOCOPHES - CZ			GC-MS/MS
	Slovenia	DEMOCOPHES - SLO			GC-MS/MS
	Luxembourg	DEMOCOPHES - LU			LC-MS/MS
	Denmark	DEMOCOPHES - DK			LC–MS/MS
	Cyprus	DEMOCOPHES - CY			LC-MS/MS
	Czech Republic	DEMOCOPHES - CZ	Cadmium		ICP-MS
	Slovenia	DEMOCOPHES - SLO			ICP-MS
	Spain	DEMOCOPHES - ES			ICP-MS
	Denmark	DEMOCOPHES - DK			ICP-MS
	Luxembourg	DEMOCOPHES - LU			ICP-MS
	Hungary	DEMOCOPHES - HU			ICP-MS
	Cyprus	DEMOCOPHES - CY			ICP-MS
	Spain	DEMOCOPHES - ES	DINCH		LC–MS/MS
	Denmark	DEMOCOPHES - DK			LC-MS/MS
	Germany	DEMOCOPHES - DE			HPLC-MS/MS
	Luxembourg	DEMOCOPHES - LU			UPLC-MS/MS
	Czech Republic	DEMOCOPHES - CZ			UHPLC-MS/MS
	Cyprus	DEMOCOPHES - CY			HPLC-MS/MS
	Czech Republic	DEMOCOPHES - CZ	Organophosphate Flame Retardants (OPFRs)		UHPLC-MS/MS
	Slovenia	DEMOCOPHES - SLO			HPLC-MS/MS
	Germany	DEMOCOPHES - DE			GC-MS/MS
	Cyprus	DEMOCOPHES - CY			LC-MS/MS
	Czech Republic	DEMOCOPHES - CZ	Phthalates	MMP, MEP, MEHP, 5OHMEHP, 5oxoMEHP, MnBP, MBzP, MCHP	UHPLC–ESI-MS/MS

				MiBP, 5CXMEHP, MECPP, MnOP, MOP, OH-MiNP, MHNP, MHiNP, cx-MiNP, MCOP, MCiOP, OH-MiDP, MHiDP, OH-MINCH, MHNCH	UHPLC-MS/MS
	Slovenia	DEMOCOPHES - SLO			UHPLC-MS/MS
	Luxembourg	DEMOCOPHES - LU			UPLC-MS/MS
	Spain	DEMOCOPHES - ES			LC–MS/MS
	Denmark	DEMOCOPHES - DK			LC–MS/MS
	Hungary	DEMOCOPHES - HU			UHPLC–ESI-MS/MS
	Cyprus	DEMOCOPHES - CY			HPLC-MS/MS
	Czech Republic	DEMOCOPHES - CZ	Polycyclic Aromatic Hydrocarbons (PAHs)		UHPLC-MS/MS
	Luxembourg	DEMOCPHES - LU			UHPLC-MS/MS
	Germany	DEMOCOPHES - DE			GC-MS
	Cyprus	DEMOCOPHES - CY			UHPLC-MS/MS
3- HBM4EU Aligned Studies	France	ESTEBAN adults-FR	Bisphenols		GC-MS/MS
	Poland	POLAES adults-PL			LC-MS/MS
	Denmark	CPHMINIPUB-DK			LC-MS/MS
	Island	Diet_HBM-IS			LC-MS/MS
	Finland	FinHealth-FI			LC-MS/MS
	Czech Republic	CELSPAC-CZ			LC-MS/MS
	Croatia	HBM in adults in Croatia-HR			LC-MS/MS
	Portugal	INSEF-ExQAP-PT			LC-MS/MS
	Switzerland	SHeS-PP-CH			LC-MS/MS
	Germany	ESB-DE			LC-MS/MS
	Luxembourg	Oriscav-Lux2-LU			LC-MS/MS
	Denmark	DYMS-DK			LC-MS/MS
	France	ESTEBAN adults-FR	Cadmium		ICP-MS
	Poland	POLAES adults-PL			ICP-MS
	Denmark	CPHMINIPUB-DK			ICP-MS
	Island	Diet_HBM-IS			ICP-MS
	Czech Republic	CELSPAC-CZ			ICP-MS
	Croatia	HBM in adults in Croatia-HR			ICP-MS
	Portugal	INSEF-ExQAP-PT			ICP-MS
	Germany	ESB-DE			ICP-MS
	Luxembourg	Oriscav-Lux2-LU			ICP-MS
	Denmark	DYMS-DK			ICP-MS
	Hungary	InAirQ-HU	DINCH		LC-MS/MS
	Italy	NACII-IT			LC-MS/MS
	Germany	GerESV children-DE			LC-MS/MS
	Norway	NEBII cildren-NO			LC-MS/MS
	France	ESTEBAN children-FR			LC-MS/MS
	Poland	POLAES children-PL			LC-MS/MS
	Slovakia	PCB children-SK			LC-MS/MS
	Slovenia	SLOCRP children-Sl			LC-MS/MS
	Greece	CROME children-EL			LC-MS/MS
	Denmark	OCC-DK			LC-MS/MS
	The Netherlands	SPECIMEN-NL			LC-MS/MS
	Belgium	3xG-BE			LC-MS/MS

Germany	GerESV children-DE	Organophosphate Flame Retardants (OPFRs)	GC-MS/MS
Norway	NEBII cildren-NO		LC-MS/MS
France	ESTEBAN children-FR		LC-MS/MS
Slovakia	PCB children-SK		LC-MS/MS
Slovenia	SLOCRP children-Sl		LC-MS/MS
Denmark	OCC-DK		LC-MS/MS
Belgium	3xG-BE		LC-MS/MS
Hungary	InAirQ-HU	Phthalates	LC-MS/MS
Italy	NACII-IT		LC-MS/MS
Germany	GerESV children-DE		LC-MS/MS
Norway	NEBII cildren-NO		LC-MS/MS
France	ESTEBAN children-FR		LC-MS/MS
Poland	POLAES children-PL		LC-MS/MS
Slovakia	PCB children-SK		LC-MS/MS
Slovenia	SLOCRP children-Sl		LC-MS/MS
Greece	CROME children-EL		LC-MS/MS
Denmark	OCC-DK		LC-MS/MS
The Netherlands	SPECIMEN-NL		LC-MS/MS
Belgium	3xG-BE		LC-MS/MS
France	ESTEBAN adults-FR	Polycyclic Aromatic Hydrocarbons (PAHs)	GC-MS/MS
Poland	POLAES adults-PL		Not specified
Denmark	CPHMINIPUB-DK		GC-MS
Island	Diet_HBM-IS		LC-MS/MS
Czech Republic	CELSPAC-CZ		LC-MS/MS
Croatia	HBM in adults in Croatia-HR		LC-MS/MS
Portugal	INSEF-ExQAP-PT		LC-MS/MS
Switzerland	SHeS-PP-CH		LC-MS/MS
Germany	ESB-DE		LC-MS/MS
Luxembourg	Oriscav-Lux2-LU		GC-MS/MS
Denmark	DYMS-DK		GC-MS

Method abbreviations: LC-MS/MS = Liquid Chromatography with tandem mass spectrometry; HPLC-ESI-MS/MS = LC-ESI-MS = Liquid Chromatography Electrospray Ionization Tandem Mass Spectrometric. ; HPLC-MS/MS = High Performance Liquid Chromatography-Mass Spectrometry; ICP-MS = Inductively Coupled Plasma Mass Spectrometry; AAS = Atomic Absorption Spectroscopy; DRC-ICP-MS = Dynamic Reaction Cell for Inductively Coupled Plasma Mass Spectrometry; LC/LC–MS/MS = Tandem Liquid Chromatography combined with Tandem Mass Spectrometry; HPLC-FD = High-performance Liquid Chromatography-Fluorescence Detector; GC-MS = Gas Chromatography–Mass Spectrometry; UHPLC-MS/MS = Ultra High Performance Liquid Chromatography-Mass Spectrometry.

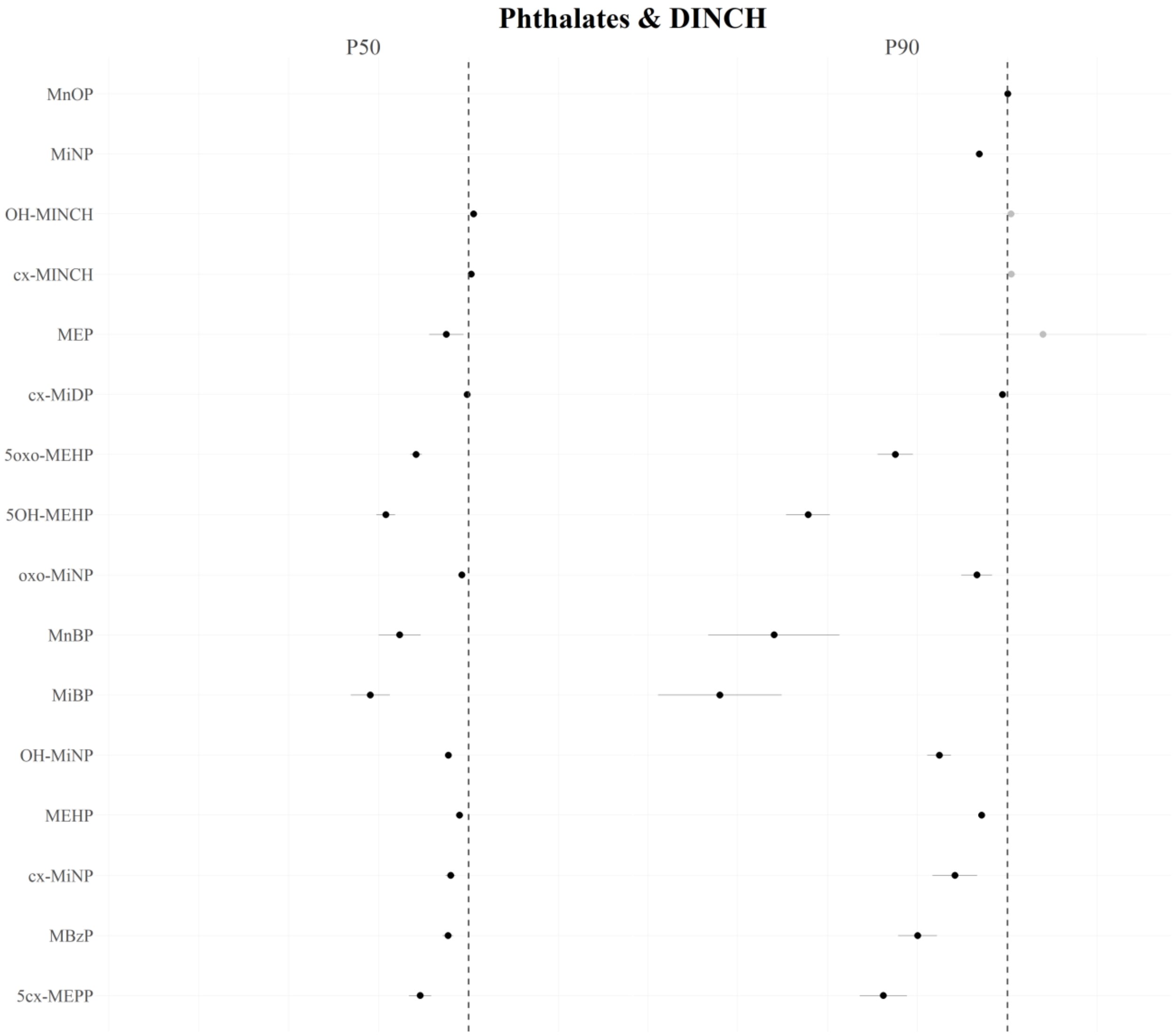

Figure S1. Forest plots representing the estimate and confidence interval for the Theil-Sen regression of phthalates and DINCH measured in children 5-12 years of age for the 50th and 90th percentiles. In black, exposure markers with p-value <0.05 (significant). In grey, exposure markers with p-value >0.05 (unsignificant).

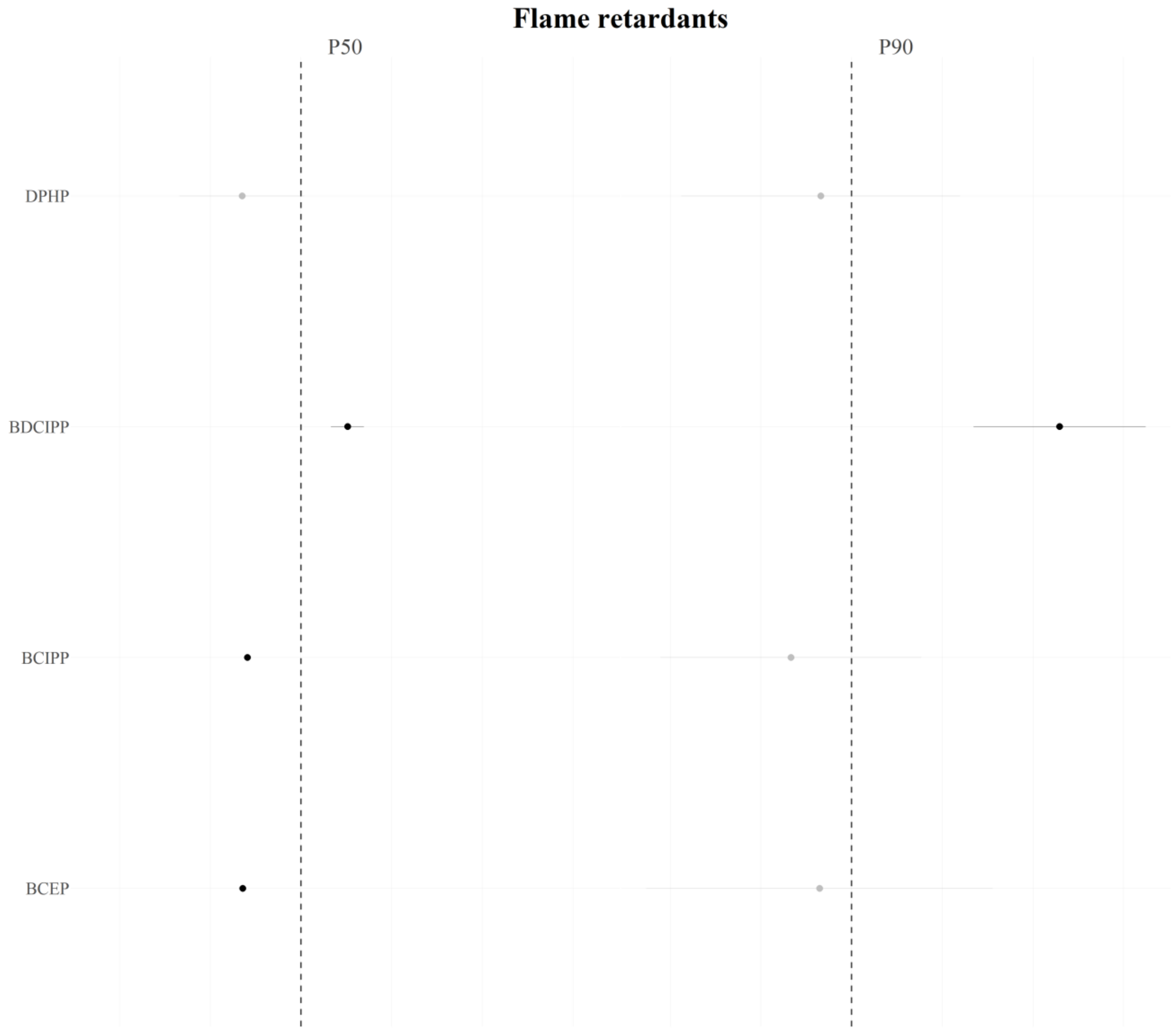

Figure S2. Forest plots representing the estimate and confidence interval for the Theil-Sen regression of OPFRs measured in children 5-12 years of age for the 50th and 90th percentiles. In black, exposure markers with p-value <0.05 (significant). In grey, exposure markers with p-value >0.05 (unsignificant).

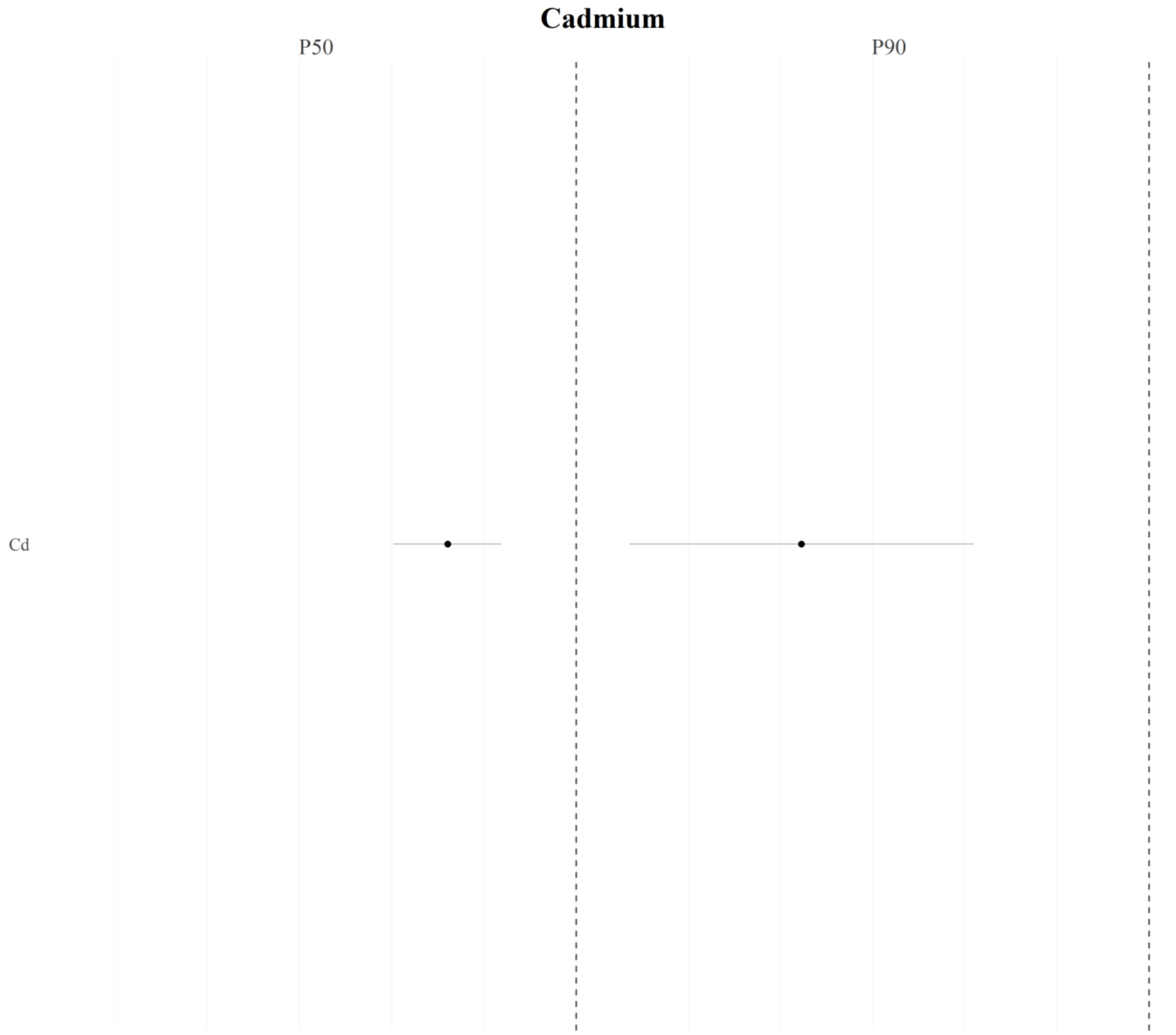

Figure S3. Forest plots representing the estimate and confidence interval for the Theil-Sen regression of cadmium measured in women 24-52 years of age for the 50th and 90th percentiles. In black, exposure markers with p-value <0.05 (significant). In grey, exposure markers with p-value >0.05 (unsignificant).

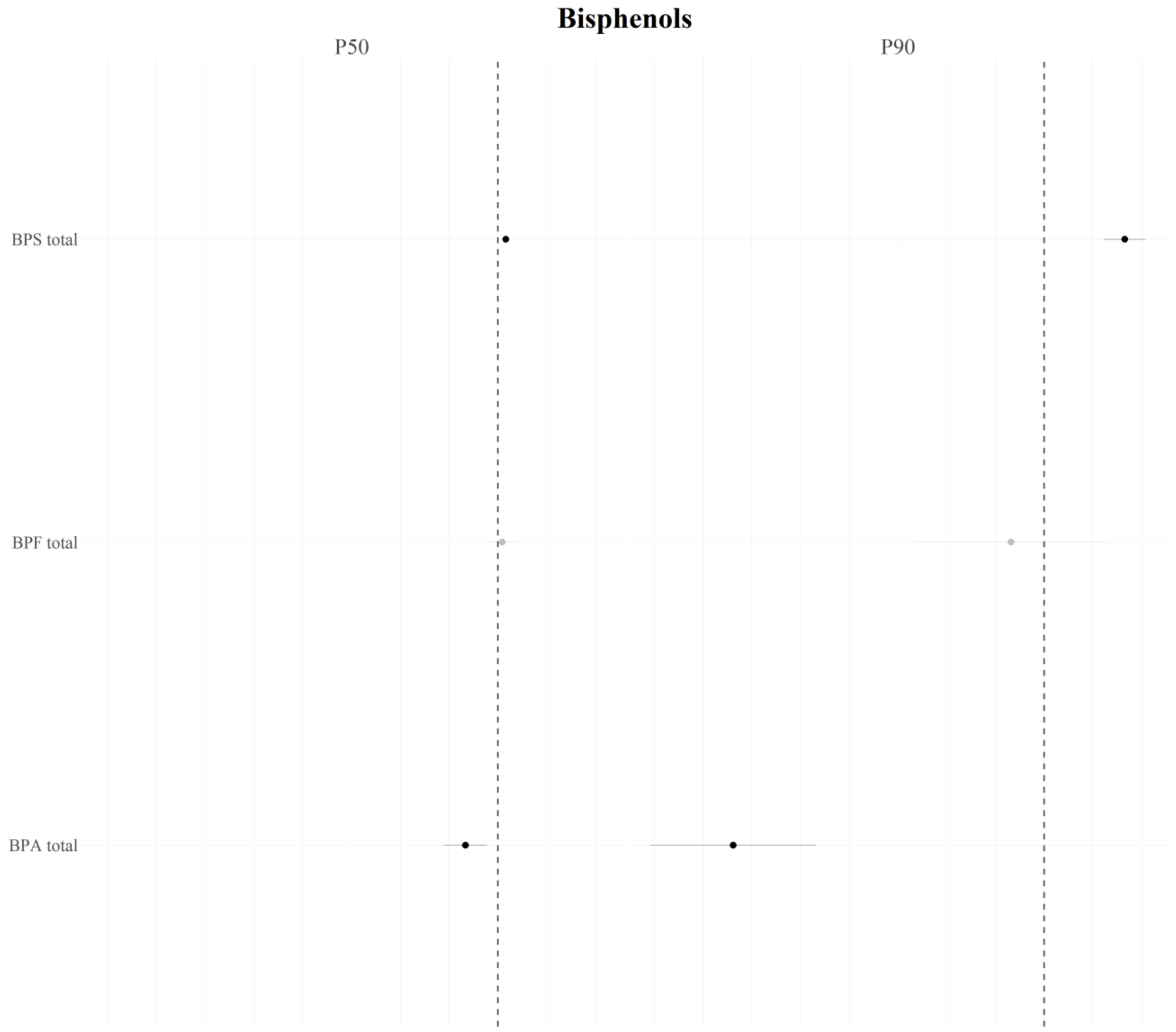

Figure S4. Forest plots representing the estimate and confidence interval for the Theil-Sen regression of bisphenols measured in women 24-52 years of age for the 50th and 90th percentiles. In black, exposure markers with p-value <0.05 (significant). In grey, exposure markers with p-value >0.05 (unsignificant).

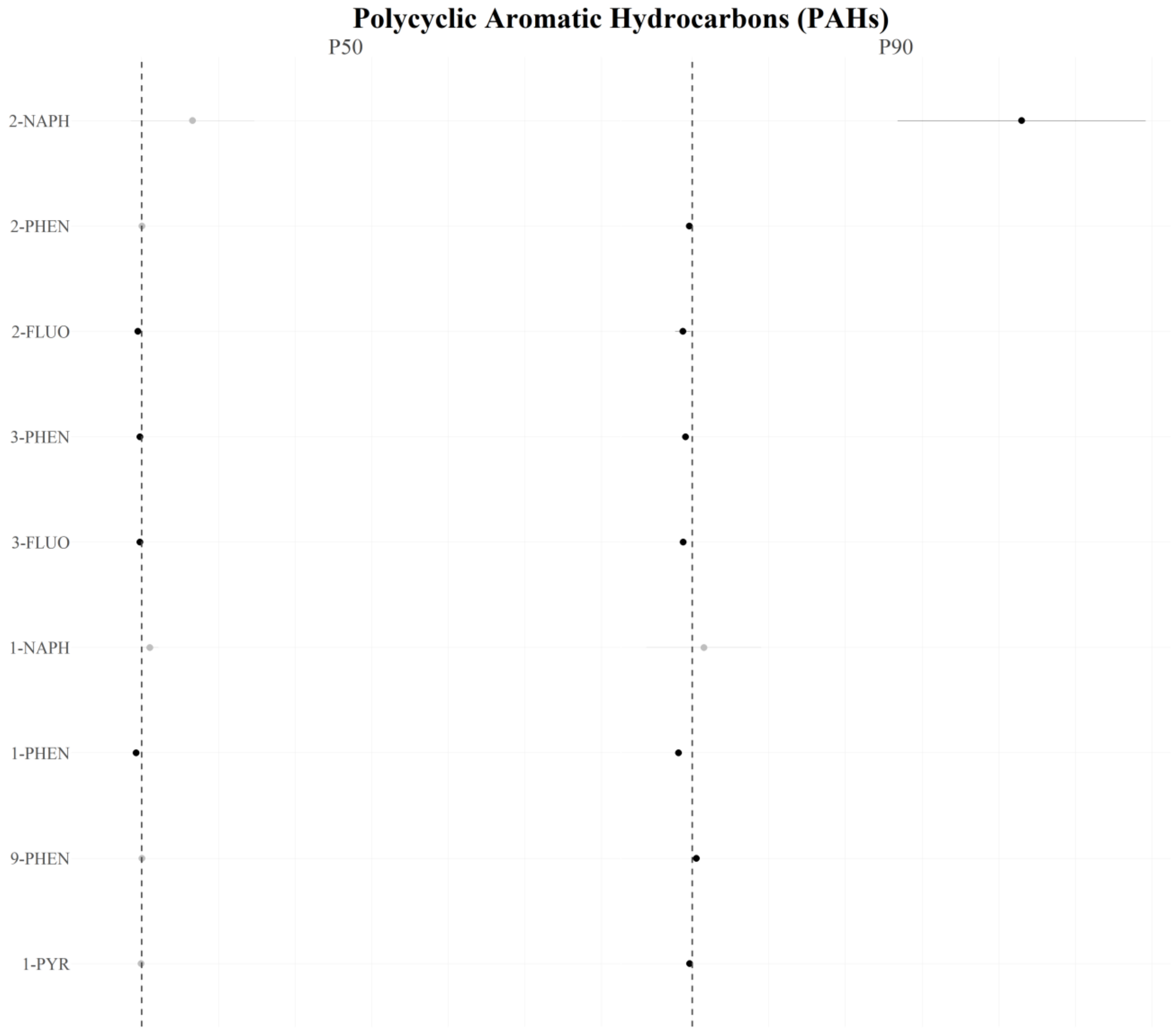

Figure S5. Forest plots representing the estimate and confidence interval for the Theil-Sen regression of PAHs measured in women 24-52 years of age for the 50th and 90th percentiles. In black, exposure markers with p-value <0.05 (significant). In grey, exposure markers with p-value >0.05 (unsignificant).
